# Genome-wide detection of somatic mosaicism at short tandem repeats

**DOI:** 10.1093/bioinformatics/btae485

**Published:** 2024-07-30

**Authors:** Aarushi Sehgal, Helyaneh Ziaei Jam, Andrew Shen, Melissa Gymrek

**Affiliations:** Department of Computer Science and Engineering, University of California San Diego, 9500 Gilman Drive, La Jolla, CA, 92093, United States; Department of Computer Science and Engineering, University of California San Diego, 9500 Gilman Drive, La Jolla, CA, 92093, United States; Department of Computer Science and Engineering, University of California San Diego, 9500 Gilman Drive, La Jolla, CA, 92093, United States; Department of Computer Science and Engineering, University of California San Diego, 9500 Gilman Drive, La Jolla, CA, 92093, United States; Department of Medicine, University of California San Diego, 9500 Gilman Drive, La Jolla, CA, 92093, United States

## Abstract

**Motivation:**

Somatic mosaicism has been implicated in several developmental disorders, cancers, and other diseases. Short tandem repeats (STRs) consist of repeated sequences of 1–6 bp and comprise >1 million loci in the human genome. Somatic mosaicism at STRs is known to play a key role in the pathogenicity of loci implicated in repeat expansion disorders and is highly prevalent in cancers exhibiting microsatellite instability. While a variety of tools have been developed to genotype germline variation at STRs, a method for systematically identifying mosaic STRs is lacking.

**Results:**

We introduce prancSTR, a novel method for detecting mosaic STRs from individual high-throughput sequencing datasets. prancSTR is designed to detect loci characterized by a single high-frequency mosaic allele, but can also detect loci with multiple mosaic alleles. Unlike many existing mosaicism detection methods for other variant types, prancSTR does not require a matched control sample as input. We show that prancSTR accurately identifies mosaic STRs in simulated data, demonstrate its feasibility by identifying candidate mosaic STRs in Illumina whole genome sequencing data derived from lymphoblastoid cell lines for individuals sequenced by the 1000 Genomes Project, and evaluate the use of prancSTR on Element and PacBio data. In addition to prancSTR, we present simTR, a novel simulation framework which simulates raw sequencing reads with realistic error profiles at STRs.

**Availability and implementation:**

prancSTR and simTR are freely available at https://github.com/gymrek-lab/trtools. Detailed documentation is available at https://trtools.readthedocs.io/.

## 1 Introduction

Population-level heterogeneity generally arises due to germline mutations that occur before the formation of the zygote and are inherited by all cells in the offspring. However, heterogeneity within an individual may also exist due to somatic mutations that occur post-zygotically in only a sub-population of cells [reviewed in Youssoufian and Pyeritz (2002)]. Somatic mosaicism has long been known to play a key role in cancer [reviewed in Stratton *et al.* (2009)], and has also been implicated in a range of nonneoplastic disorders [e.g. Proteus Syndrome (Cohen 1993), Neurofibromatosis Type 1 (Ruggieri and Huson 2001), and CLOVES syndrome (Kurek *et al.* 2012)]. Somatic mosaicism is also a hallmark of conditions resulting in DNA repair deficiencies, such as Xeroderma Pigmentosum (Cleaver 1969). Beyond its role in disease, accumulation of somatic mutations is likely a widespread phenomenon occurring in healthy individuals throughout their lifetime (Fernández *et al.* 2016).

High-throughput sequencing offers the potential to perform genome-wide detection of somatic mosaicism, but also presents important technical challenges (Dou *et al.* 2018). To distinguish somatic mutations from germline variants or technical artifacts, a matched control sample is often required to serve as a baseline. Further, in cases where the somatic mutation is present in a small fraction of cells, ultra high coverage data is needed to detect the event (Breuss *et al.* 2022). A variety of methods have been developed to address these challenges [e.g. MrMosaic (King *et al.* 2017), MosaicForecast (Dou *et al.* 2020), and DeepMosaic (Yang *et al.* 2023)]. However, existing methods in some cases still require matched control samples and focus largely on detecting mosaic single nucleotide polymorphisms (SNPs) or in some cases mosaic copy number variants [e.g. Montage (Glessner *et al.* 2021)].

Short tandem repeats (STRs), consisting of 1–6 bp sequences repeated in tandem, comprise >3% of the human genome (Lander *et al.* 2001), and exhibit rapid germline mutation rates (Sun *et al.* 2012). Somatic instability of STRs, also known as microsatellite instability (MSI), is a hallmark of certain cancers such as Lynch Syndrome [reviewed in Lynch *et al.* (2009)]. Recent work identified $10^{-4}$–$10^{-3}$ mutations per STR in non-MSI cancers, with >0.03 mutations per STR in the case of MSI (Fujimoto *et al.* 2020). In addition, somatic mutation of STRs in the brain has been implicated as a key driver of pathogenicity in some repeat expansion disorders (Swami *et al.* 2009).

Detection of somatic mosaicism at STRs from sequencing data is particularly challenging, as these regions may exhibit high error rates due to PCR artifacts (Raz *et al.* 2019) making it difficult to distinguish true somatic mutations from errors. STR-specific genotyping methods have been developed for germline genotyping that address this challenge [e.g. HipSTR (Willems *et al.* 2017) and ExpansionHunter (Dolzhenko *et al.* 2017)], but these are not designed to detect somatic events. Previous studies performed genome-wide analysis of somatic STR instability in the context of cancer (Kim *et al.* 2013, Hause *et al.* 2016, Fujimoto *et al.* 2020), but relied on comparing sequencing from tumors with matched normal samples. Further, somatic events were detected either using custom analysis pipelines not packaged as a separate tool (Kim *et al.* 2013) or were based on heuristics rather than hypothesis testing frameworks. For example (Hause *et al.* 2016), which relies on mSINGS (Salipante *et al.* 2014), identifies unstable STRs as those for which a tumor sample has one or more additional allele lengths observed compared to normal. In most cases these methods do not output well-calibrated *P*-values which can be used to control for false discovery rates, cannot incorporate locus-specific error models [although these are used in (Fujimoto *et al.* 2020)] and do not provide estimation of the length or fraction of mosaic alleles.

Here, we introduce prancSTR, a novel method for detecting mosaic STRs from high throughput sequencing data without the need for a matched control sample. prancSTR models observed reads as a mixture distribution and infers the maximum likelihood mosaic fraction and the copy number of the mosaic versus germline alleles. prancSTR is primarily designed to detect mosaicism at loci characterized by a single high-frequency mosaic allele. We expect the majority of detectable mosaic STRs from germline genomes to follow this pattern. Although they have high mutation rates compared to other variant types, it is still unlikely that multiple independent mutations would occur at the same STR locus sufficiently early to reach high enough frequency for detection. Despite this assumption, we find that prancSTR can detect cases with multiple mosaic alleles with similar power compared to single mosaic allele scenarios.

We show that prancSTR accurately identifies mosaic STRs in simulated data and validate mosaic STRs inferred from a real Illumina short read dataset with orthogonal long read data. We apply prancSTR to 460 whole genome sequencing (WGS) datasets from the 1000 Genomes Project derived from lymphoblastoid cell lines (LCLs) to characterize genome-wide mosaic STRs in different populations. Finally, we evaluate the ability of prancSTR to detect mosaic STRs across different available sequencing technologies. prancSTR can in theory be applied to data from a range of technologies, but provides most accurate results in settings with low sequencing error rates at STRs, including PCR-free Illumina and Element Biosciences data. Overall, prancSTR provides a robust method to identify mosaic STRs from existing high throughput sequencing datasets.

## 2 Materials and methods

### 2.1 prancSTR overview

#### 2.1.1 Baseline model

**Figure 1.**
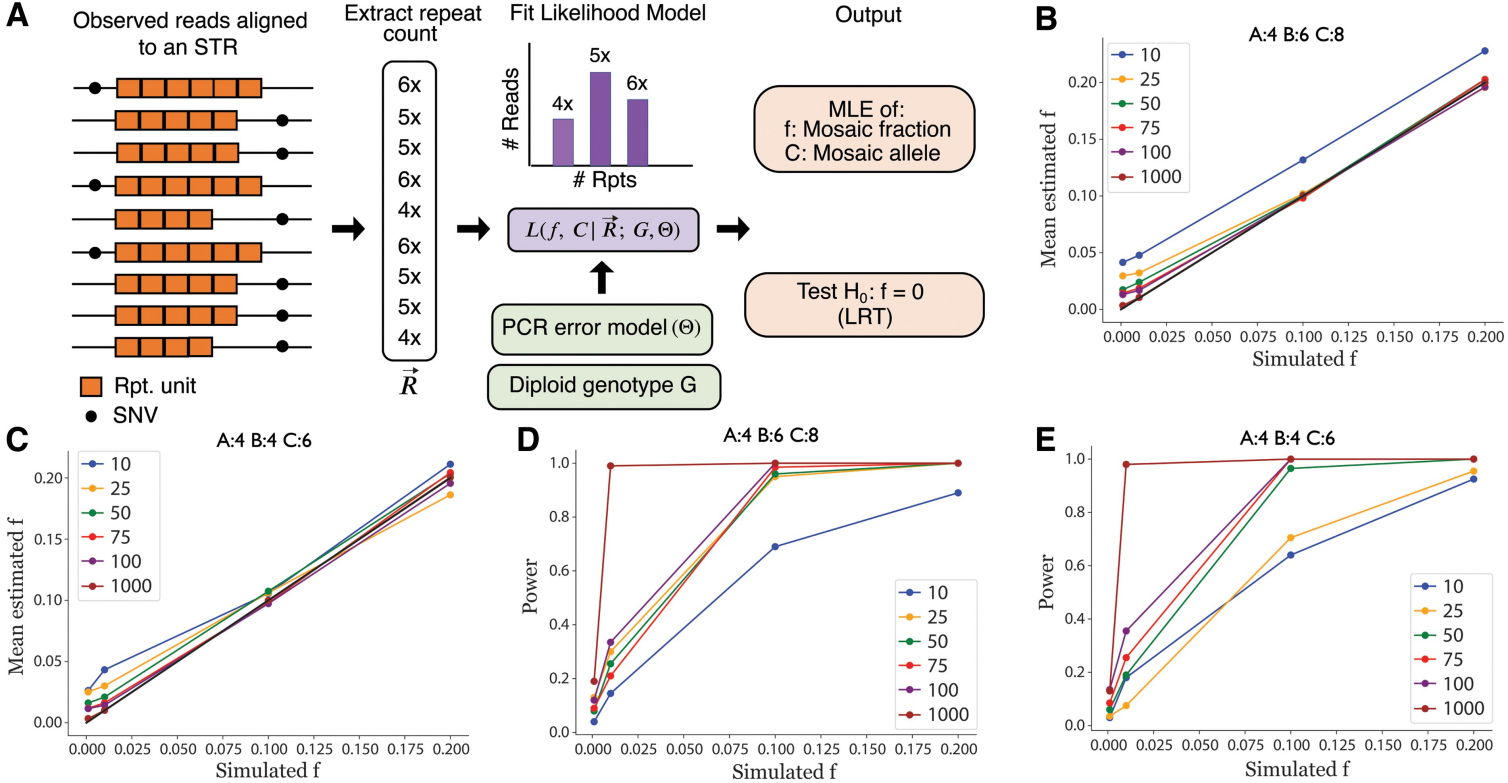
prancSTR overview and validation. (A) Overview of the prancSTR method. The copy numbers observed in each read aligned to a target STR are extracted to a vector $\vec{R}$, from which prancSTR obtains maximum likelihood estimates for the mosaic allele (*C*), mosaic allele fraction (*f*), and a *P*-value testing $H_{0}:f=0$. Single nucleotide variants (SNVs) are not directly used but are shown to illustrate that reads in each sample originate from two haplotypes. (B) and (C) Simulated versus estimated values of *f*. We simulated mosaic STRs under a range of coverage levels and values for *f* for cases in which the germline genotype is heterozygous (B) or homozygous (C). Dots represent the mean estimated *f* value from 200 simulations. The black line denotes the *x* = *y* diagonal. (D) and (E) Power to detect mosaic STRs. Power is computed as the percent of simulations for which *P *<* *0.05. For B–E, colors denote different coverage levels, where coverage gives the total number of reads spanning the STR of interest. Simulated values for germline genotypes *A*, *B*, and mosaic allele *C* are denoted at the top of each panel. Panels here are based on simulated read vectors $\vec{R}$. Similar results for simulations based on raw reads are shown in Supplementary Figs S3 and S4.

prancSTR is designed to identify mosaic STRs at one locus at a time. It takes as input STR genotypes and metadata computed by an existing genotyper and outputs candidate mosaic STRs (Fig. 1A). While designed to work downstream of HipSTR (Willems *et al.* 2017), prancSTR can theoretically process output from any STR genotyping tool as long as it returns estimated diploid repeat lengths and the observed distribution of copy numbers across all reads aligning to a locus.

At each STR locus, prancSTR takes as input a vector of the observed repeat copy number in each read, $\vec{R}=\{r_{1},r_{2},\ldots ,r_{n}\}$, where *r_i_* is the number of copies of the repeat observed in the *i*th read. For each locus, let 〈A,B〉 denote the diploid germline genotype, where *A* and *B* give the copy number of the repeat unit on each chromosome copy. Let *f* denote the fraction of chromosome copies harboring an additional allele *C* resulting from a mosaic mutation, and Θ represent additional error parameters described below. If the somatic mutation occurred on the haplotype containing allele *B*, we would expect $\frac{1}{2}$ of chromosome copies to contain allele *A*, $\frac{1}{2}-f$ to contain allele *B*, and *f* to contain allele *C*. Assuming each observed read is independent, we can then write the following likelihood equation:

(1) $$\begin{array}{c} L_{B}(C,f|\vec{R};\langle A,B\rangle ,\Theta )=\prod_{r\in \vec{R}}\frac{1}{2}S(r|A;\Theta )+\\ \left(\frac{1}{2}-f)S(r|B;\Theta )+fS(r|C;\Theta \right) \end{array}$$

where *L_B_* denotes the likelihood of *C* and *f* in the case that the mosaic allele occurred on the haplotype with allele *B*. S(r|G;Θ) gives the probability to observe *r* copies of the repeat in a read given it originated from an allele with *G* copies assuming stutter error model Θ.

#### 2.1.2 Stutter error model

The term S(r|G;Θ) is computed based on the error model used in HipSTR (Willems *et al.* 2017), which models errors in observed repeat counts in each read that arise during PCR or sequencing. The model includes three parameters. The first two model the probability for a read to contain a stutter error resulting in a repeat expansion (*u*) or contraction (*d*). The third parameter, *ρ*, models the distribution of error sizes in terms of the absolute number of repeat units. It is assumed that errors follow a geometric distribution with parameter *ρ*. The resulting equation for S(r|G;Θ) is:

(2) $$S(r|G;\Theta =\{u,d,\rho \})=\{\begin{array}{cc} 1-u-d & r==G\\ u\rho {\left(1-\rho \right)}^{r-G-1} & r>G\\ d\rho {\left(1-\rho \right)}^{G-r-1} & r<G \end{array}$$

We assume here that *u*, *d*, and *ρ* are known for each locus as these can be estimated from existing data using other methods (Willems *et al.* 2017, Kristmundsdottir *et al.* 2020).

In practice with short reads, we are unable to determine the haplotype of origin (either *A* or *B*) of the mosaic allele. Therefore below we aim to identify *C* and *f* that maximize the log likelihood over two possible cases:

(3) $$\begin{array}{c} \;\text{log}\;L(C,f|\vec{R})=\max\{\;\text{log}\;L_{A}(C,f|\vec{R}),\;\text{log}\;L_{B}(C,f|\vec{R})\} \end{array}$$

#### 2.1.3 Likelihood maximization and hypothesis testing

The goal of prancSTR is to find values for *C* and *f* that maximize Equation (3). We assume the underlying stutter model Θ and diploid genotype 〈A,B〉 are known and can be obtained from HipSTR’s output. We then use an iterative algorithm to estimate *C* and *f*:

1. Initialize the value of *f* to 0.01.
2. Compute the log-likelihood for each possible value of *C*, given *f* from step 1. We restrict our search for *C* to $\left(\min\vec{R}-3,\max\vec{R}+3\right)$. Return the value of *C* that maximizes the log-likelihood.
3. Find the value of *f* that maximizes the log-likelihood given *C* from step 2. This step is performed using Sequential Least Squares Programming (SLSQP) (Kraft 1988) restricting *f* to be between 0 and 0.5.
4. Repeat steps 2 and 3 until convergence.

In practice, the read vector $\vec{R}$ is obtained from the MALLREADS format field from HipSTR VCF files. We exclude STR calls from analysis if: they have coverage of 0, have missing genotypes, have 0 read support in MALLREADS for the called diploid genotype, or if there is only evidence in MALLREADS of reads from a single allele.

After obtaining the maximum likelihood estimates $\hat{f}$ and $\hat{C}$, prancSTR computes the likelihood ratio test statistic *λ_LR_*:

(4) $$\lambda_{LR}=-2\ln\frac{L(\hat{C},f=0|\vec{R};\langle A,B\rangle ,\Theta )}{L(\hat{C},\hat{f}|\vec{R};\langle A,B\rangle ,\Theta )}$$

Finally, we use the likelihood ratio statistic to obtain a *P*-value testing $H_{0}:f=0$ at each STR in each sample. Typically, this statistic is expected to follow a $\chi^{2}$(2) distribution under the null. However, the null hypothesis (*f *=* *0) falls on the boundary of the parameter space, which violates an assumption of the likelihood ratio test. Following a previously published method, we use a null consisting of a mixture distribution of a point mass at 0, with 50% probability, and a chi-square distribution with 2 degrees of freedom, also with 50% probability (Nielsen and Wakeley 2001). Although that method assumes a single parameter of interest whereas we are estimating two (*C* and *f*), our simulations suggest the resulting *P*-values are generally well calibrated (Supplementary Fig. S1).

### 2.2 Simulating vectors of observed repeat counts

In our first simulation strategy, we simulated vectors of observed repeat counts for a single locus under various parameter settings. Although prancSTR assumes a single mosaic allele, our framework enables simulating 0 or more mosaic alleles to evaluate a diverse range of settings. In each case, we model *k* mosaic alleles $C_{1}\ldots C_{k}$, their corresponding allele fractions $f_{1}\ldots f_{k}$, germline allele A with frequency 0.5 and germline allele B with frequency 0.5–$\sum_{i}f_{i}$, requiring the total frequencies of A, B, and all mosaic alleles to sum to 1. The resulting read count vectors, as well as the known values of *A*, *B*, and Θ, were used as input to prancSTR’s likelihood estimation procedure.

For each tested setting, we performed 200 simulations. Power was estimated as the percentage of simulation rounds for which prancSTR returned a significant *P*-value (*P* < 0.05). Notably this captures relative power differences across settings but is not reflective of the absolute power in genome-wide analyses, in which a more stringent *P*-value threshold is required to account for multiple hypothesis testing. To evaluate false positive rates, we performed simulations with *f* set to 0 and similarly returned the percentage of simulation rounds with significant *P*-values.

### 2.3 A method for simulating error-prone next-generation sequencing reads at STRs

For our second simulation strategy, we developed a novel simulation framework, simTR, which simulates raw sequencing reads according to a specified coverage level and error model using user-defined repeat alleles. simTR is a wrapper built around ART (Huang *et al.* 2012), an existing, open source, next generation sequencing read simulator. ART creates simulated reads that account for generic insertion and deletion mutations. However, stutter errors (additions or deletions of one or more repeat units introduced during PCR) characteristic of STRs are not specifically modeled. simTR adds to ART by incorporating stutter errors into the simulated reads, in addition to existing indel mutations. Stutter errors are incorporated based on the HipSTR error model described in Equation (2).

simTR takes as input a genome file (fasta format), the genomic coordinates of the target STR, and stutter parameters (*u*, *d*, and *ρ*). Users may also specify optional parameters to set the desired coverage, to generate paired-end versus single-end reads, the mean and standard deviation of the sequencing fragment lengths, and the window size around the STR from which to simulate reads. It creates intermediate fasta files with separate entries to represent the different possible observed repeat lengths that could result from PCR stutter. It then invokes ART to simulate reads from the different fasta entries at rates proportional the expected proportion of each allele based on the input stutter parameters. Finally, it outputs simulated reads in fastq format which can be used for benchmarking downstream tools.

To evaluate the entire prancSTR pipeline starting from raw reads, we applied simTR to perform *in silico* titration experiments in which we simulate reads at a target set of mosaic STRs under a range of settings by targeting various coverage levels for the germline and mosaic alleles to mimic different mosaic frequencies. Simulated reads were aligned to a reference genome (hg38) using BWA MEM (Li 2013) version 0.7.12-r1039. The resulting reads were used as input to HipSTR v0.6.1 for genotyping the target STRs using non-default options min-reads 5 and stutter-in to provide a file with simulated stutter error parameters. The VCF output by HipSTR was then used as input to prancSTR to estimate *C* and *f*. An example IGV (Thorvaldsdóttir *et al.* 2013) screenshot for simulated reads at a mosaic STR is shown in Supplementary Fig. S2.

### 2.4 Characterizing mosaic STRs in the 1000 Genomes Project

We focused on individuals from the 1000 Genomes Project dataset belonging to CEU (Northern Europeans from Utah; *n* = 179), YRI (Yorubans from Nigeria; *n* = 178), and CHB (Han Chinese; *n* = 103) populations for which high-coverage PCR-free WGS is available (Byrska-Bishop *et al.* 2022). We applied prancSTR to identify candidate mosaic STRs in these samples based on previously obtained HipSTR calls (Ziaei Jam *et al.* 2023). These calls had already been filtered to exclude loci with call rate <75%, loci with genotypes not matching Hardy-Weinberg expectation (*P* < 1e−06), and loci overlapping segmental duplications in the human genome. prancSTR output was filtered to include candidate mosaic STRs with: at least three reads supporting the identified mosaic allele *C*, read depth at least 10, with f≤0.3 (larger *f* indicates likely heterozygous sites), HipSTR quality score ≥0.8. To adjust for multiple hypothesis correction, we applied the Benjamini–Hochberg (Benjamini and Hochberg 1995) method to identify mosaic STRs at a false discovery rate of 5%.

Hard to map regions of the genome (Olson *et al.* 2022) were obtained from https://ftp-trace.ncbi.nlm.nih.gov/ReferenceSamples/giab/release/genome-stratifications/v3.3/GRCh38https://ftp-trace.ncbi.nlm.nih.gov/ReferenceSamples/giab/release/genome-stratifications/v3.3/GRCh38@all/Union/GRCh38_alllowmapandsegdupregions.bed.gz.The intersectBed utility of BEDTools (Quinlan and Hall 2010) v2.28.0 was used to intersect STR coordinates with these regions. For downstream analyses, we removed mosaic STRs identified in >10 samples in a single population and those overlapping hard to map regions.

Samples with outlier numbers of mosaic STRs were identified as those with counts more than two standard deviations above the mean across all individuals in each population. WGS sequencing coverage and EBV coverage for each sample was obtained from the 1000 Genomes Project website: http://ftp.1000genomes.ebi.ac.uk/vol1/ftp/data_collections/1000G_2504_high_coverage/1000G_2504_high_coverage.sequence.index and http://ftp.1000genomes.ebi.ac.uk/vol1/ftp/technical/working/20130606_sample_info/20130606_sample_info.txt.

### 2.5 Validating mosaic STRs from NA12878 using PacBio HiFi long reads

Aligned reads (BAM) for NA12878 based on PacBio HiFi long reads were obtained from Genome In A Bottle (https://ftp-trace.ncbi.nlm.nih.gov/ReferenceSamples/giab/data/NA12878/PacBio_SequelII_CCS_11kb/HG001_GRCh38/). We used the haplotag (HP) tag to partition the BAM into separate files containing reads for each haplotype. We then used HipSTR v0.7 to separately genotype reads from each haplotype using the following non-default parameters to enable running HipSTR on long reads: def-stutter-model, max-str-len 1000, max-flank-indel 1, use-unpaired, no-rmdup, min-reads 5, output-filters. We extracted the MALLREADS field from the HipSTR VCF file to examine support for each allele in PacBio reads for each haplotype. Analysis was restricted to loci with at least 10 spanning PacBio reads from each of the two haplotypes.

For comparison, we performed a similar analysis on all CEU samples, including NA12878, to assess how often PacBio reads (from NA12878) would appear to validate a mosaic STR that was identified in a different sample. For this analysis, we further filtered: (i) mosaic STRs where the mosaic allele was found on >80% of PacBio reads from a single haplotype (indicating the mosaic allele for a sample was most likely the NA12878 germline allele) and (ii) unique STR loci identified as mosaic STRs in >5 samples, indicating the locus is potentially problematic. Finally, we excluded samples from the comparison if fewer than five sites remained in a particular category, as the metrics computed are unreliable on low count numbers.

### 2.6 Evaluation of prancSTR on different sequencing technologies

High-coverage (100×) PCR-free Element data (2 × 150bp) for sample HG001 (NA12878) was obtained from s3://element-public-data/2023-Cloudbreak/adept_human_wgs/bases2fastq/APP-1419/GAT-LI-C014/Elembio-2023-Cloudbreak/APP-1419/HG001-cb-500bp__GAT-LI-C014/. Reads were aligned to the GRCh38 reference genome using bwa mem (Li 2013) version 0.7.12-r1039 and converted to sorted and indexed BAM format using samtools (Li *et al.* 2009) version 1.9. STRs were genotyped in both datasets using HipSTR (Willems *et al.* 2017) with its GRCh38 reference panel. For both datasets, we used non-default parameters bam-samps HG001 bam-libs HG001, def-stutter-model, no-rmdup, min-reads 5, output-filters. For PacBio data, we additionally used the options use-unpaired, max-str-len 1000, max-flank-indel 1. HipSTR results for autosomal regions were filtered with dumpSTR (Mousavi *et al.* 2021) v6.0.2 to remove calls with <10 supporting reads, quality score <0.8, and loci overlapping segmental duplications in hg38 [obtained from the UCSC Genome Browser (Kent *et al.* 2002)].

To fit technology-specific error models, we wrote a custom pipeline that learns a single stutter error model for each repeat unit length by jointly analyzing observed reads across all STRs with that unit length. We first extracted loci for which Illumina genotypes were confidently (Q > 0.9, DP > 10) called as homozygous in NA12878. We then assume that all observed reads not matching the called allele at those loci are due to errors. We annotated each observed read as containing an insertion error, deletion error, or match, compared to the called allele and recorded the absolute difference in the number of repeat units compared to the called allele for each read. We inferred stutter parameters as: *u* =(num insertion reads/num total reads) and *d* =(num deletion reads/num total reads), and *ρ* =(percentage of reads with an error size of 1 unit), noting that under the geometric model used for step sizes *ρ* is equivalent to this percentage. Before estimating *ρ*, we excluded reads with extreme step sizes (>10) as these are unlikely to be due to stutter error. For comparison, we applied the same script to Illumina data for NA12878 to ensure similar stutter parameters were obtained compared to those output by HipSTR.

Filtered VCF files along with technology-specific stutter error models were used as input to prancSTR with option only-passing to process only loci passing dumpSTR’s quality filters. Mosaic STRs identified by prancSTR were filtered to include only those with: adjusted *P* < 0.05, minimum mosaic support 3 reads, minimum total coverage 10 reads, maximum mosaic allele fraction 0.3, genotype quality score ≥0.8. We further filtered mosaic STRs from PacBio HiFi results for which reads showed evidence for >5 distinct alleles and that had extremely high coverage (>100×). We considered a mosaic STR to be replicated across technologies if the same locus passed the above criteria in results from each one, even if reported values of *C* and *f* differed.

## 3 Results

### 3.1 Benchmarking prancSTR using simulated data

To evaluate prancSTR, we performed simulations using two strategies (Section 2). First, to evaluate our likelihood maximization procedure, we simulated vectors of observed repeat counts in each read aligned to a locus ($\vec{R}$) according to the baseline model described in Section 2. In this case, we assumed the germline (diploid) genotype 〈A,B〉 is known, and used the ground truth values of *A* and *B* as well as the simulated read vectors as input to the maximum likelihood estimation of mosaic allele (*C*) and mosaic fraction (*f*). Second, to evaluate our end to end pipeline starting from raw reads, we used simTR to simulate reads for mosaic STRs under a range of conditions, which were used as input to HipSTR to infer the germline genotype and compute read vectors. HipSTR results were used as input for mosaicism detection.

We first evaluated prancSTR under the null setting of *f *=* *0 to determine how often we falsely detect a significant mosaic STR. *P*-values returned by prancSTR are well-calibrated, following the expected uniform distribution in this case (Supplementary Fig. S1A and B). As expected, at a *P*-value threshold of 0.05, prancSTR falsely identifies approximately 5% of null simulation rounds as significant mosaic STRs in the case of error rates similar to PCR free Illumina data (Supplementary Fig. S1C and D). False positive rates are slightly increased when simulating data under PCR+ conditions (Supplementary Fig. S1E and F). As expected, false positive rates are dramatically higher when running prancSTR with error model parameters that do not match the input data (e.g. using PCR-free models for PCR+ data; Supplementary Fig. S1G and H).

Next, we simulated mosaic STRs under a range of values for coverage and mosaic allele fraction and for cases in which the germline genotype is either homozygous or heterozygous. Using both simulation strategies, estimated values of the mosaic allele fraction $\hat{f}$ are highly consistent with simulated values (Fig. 1B and C, Supplementary Figs S3 and S4). In cases that are underpowered (*f *<* *0.02 and/or coverage 10×), prancSTR tends to slightly but consistently overestimate the mosaic allele fraction. In practice, these cases are unlikely to reach genome-wide significance.

As expected, power to detect mosaic STRs increases as a function of *f* and sequencing coverage in all simulation settings (Fig. 1D and E, Supplementary Figs S3 and S4) with near perfect power at *P* < 0.05 to detect mosaic STRs with *f *>* *0.1 at loci with at least 50× coverage. In both simulation strategies, power is higher when the germline genotype is heterozygous versus homozygous. This difference is more pronounced in results based on simTR simulations. In that case, this bias is partially explained by genotyping errors. We observed that cases where the simulated germline genotype is homozygous but the mosaic fraction is high are consistently misidentified by HipSTR as heterozygous sites, and therefore cannot be identified by prancSTR as mosaic STRs. We additionally evaluated the impact of the mosaic allele size on power. We observed that power increases with the absolute difference in length of the mosaic allele compared to the nearest germline allele (Supplementary Fig. S5). This is expected, since larger differences in size make it easier to distinguish true mosaic alleles from errors.

We next evaluated the impact of sequencing errors at STRs on the ability to detect mosaic STRs from simulated read vectors under varying stutter model parameters meant to capture typical error rates in PCR+ (∼10% of reads) versus PCR-free (∼1% of reads) data (Supplementary Figs S6–S8). As expected, with high stutter error rate, power is reduced in cases of low coverage and low mosaic fraction, and estimates of *C* and *f* show greater variability. This suggests detection of mosaic STRs will perform poorly on PCR+ short read data, where stutter error rates may often exceed expected mosaic fractions.

Finally, we evaluated the ability of prancSTR to detect mosaic STRs in cases where multiple mosaic alleles are present. In the first setting, we simulated 2–3 mosaic alleles (total mosaic fraction ranging from 0.03 to 0.3) for a range of coverage and frequency values (Supplementary Fig. S9). In the second, we simulated a spread of larger allele lengths to represent patterns of mosaicism seen in repeat expansion disorders (Swami *et al.* 2009) (Supplementary Fig. S10). In both cases, we found that prancSTR has high power to detect mosaicism under similar settings where we were powered to detect a single mosaic allele: coverage at least 50×, mosaic fraction >0.1, with increased power when the mosaic alleles show larger differences from the germline alleles. By design, prancSTR will still only return a single inferred mosaic allele and fraction (*f*) in these cases. We found that the inferred *f* tended to match either the sum of the fraction of all mosaic alleles (in cases where the mosaic alleles were similar to each other) or the fraction of the most frequent mosaic allele (in cases where the mosaic alleles showed greater differences; Supplementary Figs S9 and S10). Overall, these results suggest that although prancSTR’s model assumes a single mosaic allele, it has reasonable power to detect mosaic STRs even in cases where this assumption is broken.

### 3.2 Population-wide characterization of mosaic STRs

We next applied prancSTR to characterize population-wide trends of STR mosaicism. We focused on individuals from 1000 Genomes samples from the CEU (Northern Europeans from Utah; *n* = 179), YRI (Yorubans from Nigeria; *n* = 178), and CHB (Han Chinese; *n* = 103) populations for which high-coverage PCR-free WGS is available (Byrska-Bishop *et al.* 2022). Notably, since the data is LCL-derived, identified mosaic STRs may consist of a combination of true somatic mutations that existed before sample collection as well as mutations that have accumulated during cell line passages, a phenomenon that has been previously observed for STRs and structural variants (Mohyuddin *et al.* 2004, Scheinfeldt *et al.* 2018).

**Figure 2.**
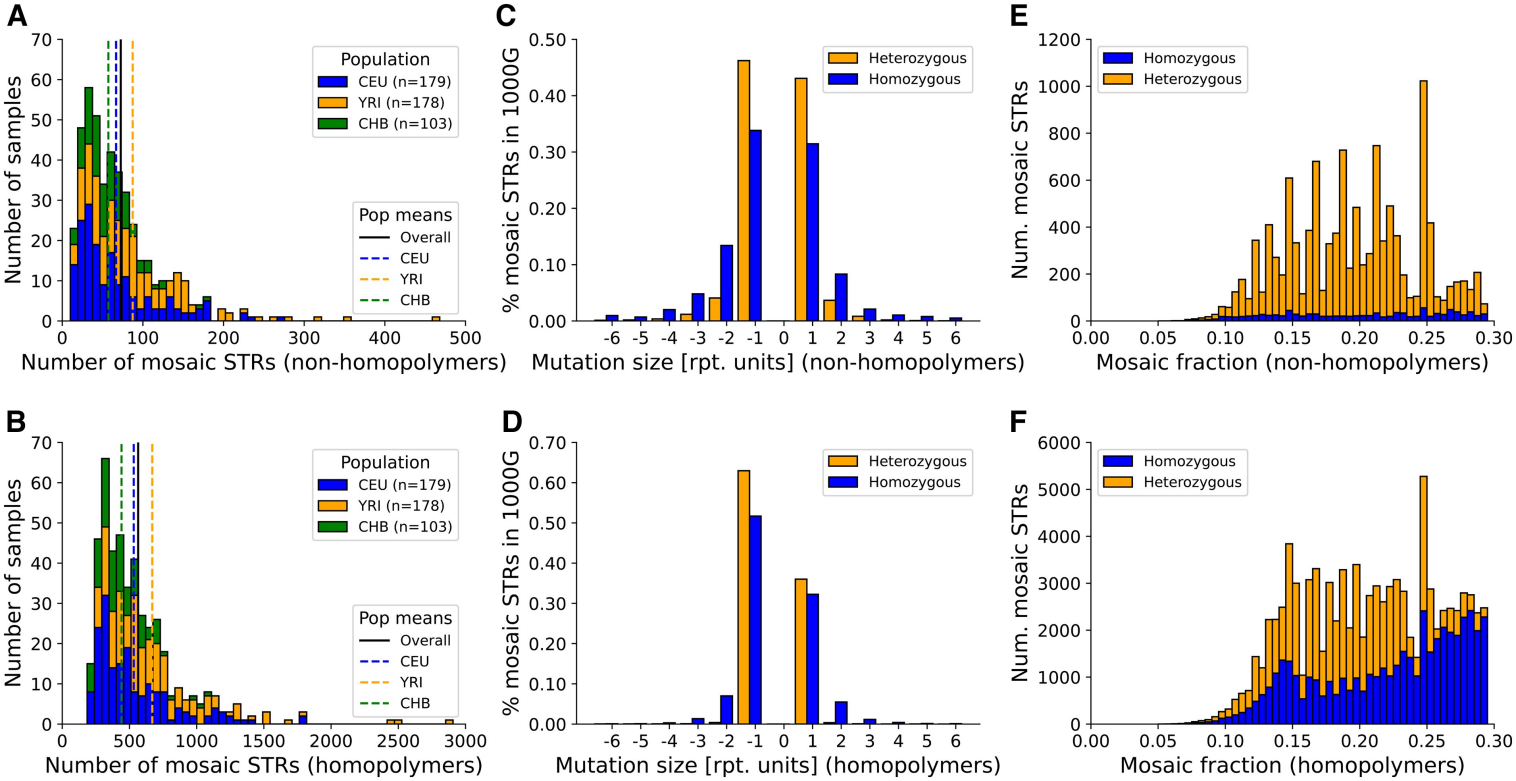
Mosaic STR trends across populations in the 1000 Genomes dataset. (A) and (B) Distribution of the number of mosaic STRs across different populations. The *x*-axis gives the number of mosaic STRs for a given population and the *y*-axis gives the number of samples. Data is shown for nonhomopolymers (A) and homopolymers (B). Dashed colored lines (CEU=blue, YRI=orange, CHB=green) give population-specific means, and the black line denotes the overall mean. Bars are stacked. (C) and (D) Distribution of mosaic STR mutation sizes. The *x*-axis represents the mutation size, computed as the difference between the mosaic allele length and the closest germline allele. Positive mutation sizes indicate insertions and negative sizes indicate deletions. Data is shown for nonhomopolymers (C) and homopolymers (D) and is for CEU only. Other populations showed similar trends. Blue represents homozygous loci and orange represents heterozygous loci. (E) and (F) Distribution of mosaic allele fraction (*f*) across mosaic STRs. Data is shown for nonhomopolymers (E) and homopolymers (F) and is for CEU only. Bars are stacked and colored to denote the number of mosaic STRs occurring at homozygous (blue) versus heterozygous (orange) sites.

A total of 84,373, 103,473, and 45,682 unique mosaic STRs were identified in the CEU, YRI, and CHB populations. Example visualizations of read alignments spanning mosaic STRs are shown in Supplementary Fig. S11. Mosaic STRs were identified across all autosomal chromosomes (Supplementary Fig. S12). As expected, the majority of unique mosaic STRs (range 75.7%–86.6% in each population) were only identified in a single sample in each population. A small number of mosaic STRs (range 41–121 in each population) were identified in >10 individuals in a single population. Further, we found that mosaic STRs are enriched in difficult to map regions (Fisher Exact two-sided $P<10^{-91}$ in all groups, mean OR = 1.8). These highly recurrent mosaic STRs and those falling in challenging regions of the genome are likely enriched for false positive calls and thus were filtered from downstream analyses. After filtering these loci and performing additional quality filtering (Section 2), 81,710, 100,407, and 44,129 unique mosaic STRs remained in CEU, YRI, and CHB. On average we identified 73 (565) nonhomopolymer (homopolymer) mosaic STRs per cell line (Fig. 2A and B) corresponding to a rate of 0.00072 (0.00191) mosaic STRs per nonhomopolymer (homopolymer) tested. Overall, homopolymers far outnumber nonhomopolymers among mosaic STRs, and the majority of mosaic nonhomopolymers identified occur at loci for which the germline genotype is heterozygous (Supplementary Fig. S13). These trends are consistent across all populations analyzed.

We noticed substantial variation in mosaic STR counts across cell lines. The number of homopolymer and nonhomopolymer mosaic STRs per cell line are highly correlated (Supplementary Fig. S14), with the correlation strongest when considering mosaic STR calls at germline heterozygous sites (Pearson *r* = 0.97, two-sided *P* = 1.96e−110 in CEU). We also identified 13, 8, and 4 cell lines from CEU, YRI, and CHB, respectively, with outlier total mosaic STR counts (Section 2). Overall, these results suggest certain cell lines have higher rates of STR instability, potentially due to genetic or environmental factors. Variation in mosaic STR counts across cell lines is not significantly correlated with the number of sites considered or EBV virus count (two-sided *P*≥ 0.05), and is only modestly correlated with sequencing coverage (Pearson *r* = 0.20, two-sided *P* = 0.051 for nonhomopolymers and *r* = 0.16, *P* = 0.10 for homopolymers; Supplementary Fig. S15). Passage numbers for these cell lines were not available, and so the impact of cell culture history, which is likely to play a role in mutation counts, could not be assessed.

We next investigated the distribution of the sizes of mosaic STR mutations. The majority of events (86.9% and 90.8% for nonhomopolymer and homopolymer mosaic STRs, respectively) result in insertions or deletions of a single repeat unit (Fig. 2C and D), although larger step sizes were observed. Mutation sizes are larger on average for mutations at STRs with homozygous versus heterozygous germline genotypes and show an overall bias toward deletions versus contractions. A similar deletion bias has been observed for somatic mutations at STRs in cancer (Fujimoto *et al.* 2020). However, both biases described above are more pronounced at homopolymer loci, suggesting they may arise in part from erroneous mosaic STR calls (Supplementary Fig. S16). Indeed, inferred stutter error rates suggest deletion errors are more common than insertions (Supplementary Figs S7 and S8), and large mutation step sizes at homozygous sites may reflect true heterozygous sites that were incorrectly genotyped.

We then examined the distribution of variant allele fractions (*f*) for detected mosaic STRs (Fig. 2E and F, Supplementary Fig. S17). In all cases, *f* distributions show peaks around 0.15–0.20, consistent with the range where we expect to have sufficient power (Fig. 1D and E), whereas true mosaic sites with higher *f* values are likely to be indistinguishable from heterozygous sites. Further, homopolymer calls with high *f* values nearly all occur at homozygous sites, and the observed deletion bias is strongest overall for sites with high *f* values, indicating mosaic STRs with *f *>* *0.2 may be enriched for false positive calls.

### 3.3 Validating mosaic STRs in NA12878

To further evaluate whether our pipeline is identifying true mosaic STRs, we performed a more detailed analysis of mosaic STRs identified in the highly characterized NA12878 sample. To evaluate these loci, we compared to an orthogonal dataset of haplotagged PacBio HiFi long reads (mean coverage ∼30×) available for the same individual (Section 2). Notably, although PacBio HiFi shows high accuracy at most regions, it has elevated error rates at homopolymers (Wenger *et al.* 2019), suggesting repeat counts obtained from PacBio reads at those loci may not serve as an accurate ground truth dataset. In addition, we observed that inferred stutter error rates in short reads are highest at homopolymer STRs (Supplementary Fig. S7). Therefore, results below are reported separately for nonhomopolymer versus homopolymer STRs.

**Figure 3.**
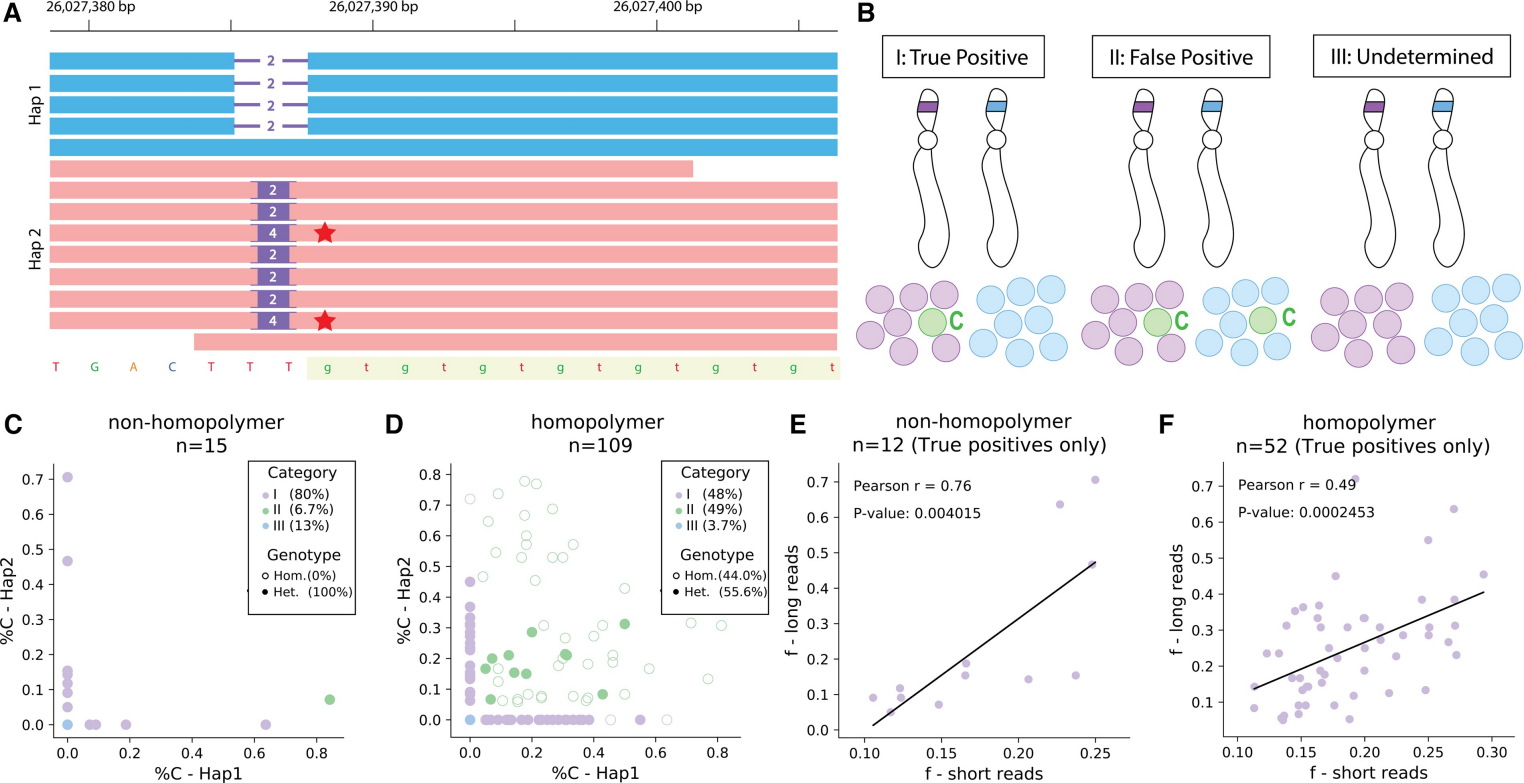
Validating candidate mosaic STRs identified in short reads from NA12878 (HG001) with PacBio HiFi reads. (A) Schematic representation of mosaicism validation with long reads. PacBio HiFi reads are haplotagged as belonging to either of the two haplotypes in a sample. The highlighted region denotes the STR region. Horizontal lines indicate deletions and purple rectangles indicate insertions compared to the reference genome. Mosaic alleles (red star) are typically expected to occur on only one of the two haplotypes. (B) Visualization of categories for validation of mosaic STRs in long read data. We classified mosaic STRs into several categories for long read data validation: I: Potential true positives (mosaic allele observed only on a single haplotype), II: Potential false positives (mosaic allele observed on both haplotypes) and III: Undetermined (mosaic allele not observed on either haplotype). The germline alleles are represented by purple and blue and the mosaic allele is represented by green. (C) and (D) Mosaic allele support in long reads from each haplotype. The *x*-axis and *y*-axis show the percentage of reads on each haplotype matching the mosaic allele for nonhomopolymer (C) and homopolymer (D) loci. Mosaic STRs were classified according to the three categories described above (purple=potential true positives, green=potential false positives, blue=undetermined). (E) and (F) Correlation of mosaic fraction between short and long reads. Comparisons of estimated allele fractions (for the mosaic allele identified in short reads) from short reads (*x*-axis) versus PacBio reads (*y*-axis) are shown for nonhomopolymer (E) and homopolymer (F) mosaic STRs. Black lines denote the best fit line. Only mosaic STRs categorized as potential true positives are included.

We reasoned that true mosaic alleles with sufficiently high variant allele fractions should be observed in both datasets, and that the mosaic allele should typically only occur on long reads from one of the two haplotypes at a locus (Fig. 3A). On the other hand, inferred mosaic alleles that are actually due to stutter or other error sources might be found on both haplotypes. After filtering, our analysis above had identified 411 candidate autosomal mosaic STRs in NA12878, 375 (91%) of which occur at homopolymer loci. Of these, we deemed 15 (109) corresponding to 42% (29%) of candidate nonhomopolymer (homopolymer) mosaic STRs to have sufficient PacBio HiFi coverage (at least 10 reads per haplotype) to attempt validation.

For each candidate mosaic STR, we examined the percentage of long reads from each haplotype supporting the inferred mosaic allele (*C*) (Fig. 3B–D) and classified calls into three categories. Likely true positives, corresponding to 80% (48%) of nonhomopolymers (homopolymers), consist of calls for which *C* is only identified in HiFi reads from a single haplotype. For these sites, variant allele fractions estimated from short reads are strongly correlated with those observed in the HiFi reads (Pearson *r* = 0.76, two-sided *P* = 0.0040 for nonhomopolymers and *r* = 0.49, *P* = 0.00025 for homopolymers; Fig. 3E and F). Likely false positives, corresponding to 6.7% (49%) of nonhomopolymers (homopolymers), consist of calls for which *C* is supported by at least one HiFi read from each haplotype. Undetermined, corresponding to 13% (4%) of nonhomopolymers (homopolymers), consists of calls for which *C* is not supported by long reads on either haplotype. This could indicate an incorrect mosaic STR call, but could also originate from insufficient coverage at mosaic STRs with low variant allele fractions.

We further examined read support on each haplotype at remaining candidate mosaic STRs (Supplementary Fig. S18). This revealed that the majority of homopolymer mosaic STRs identified as likely false positives occurred at loci for which the germline genotype was called as homozygous and long reads from both haplotypes supported multiple different alleles, suggesting reads at these loci are error prone. We additionally observed across all loci that the majority of validated high-confidence mosaic STRs occur at loci for which the germline genotype is heterozygous. This is consistent with our simulation results, in which true mosaic alleles with high mosaic allele fraction occurring at homozygous sites are incorrectly genotyped as heterozygous and therefore systematically missing from our mosaic STR callset. On the other hand, those with low allele fraction are unlikely to be detected at genome-wide significance. Overall, in combination with the population-wide analysis performed above, our results suggest mosaic STRs identified at heterozygous sites at moderate *f* values are robust, whereas accurate identification of mosaicism at homopolymers or for sites with either high or very low mosaic allele fractions remains challenging with short read data.

We evaluated whether mosaic STRs detected from short reads for NA12878 may appear to validate in HiFi data simply due to the high indel error rates in long reads, which could result in reads that match the inferred mosaic just due to chance. To test this, we determined how often mosaic STRs inferred from short reads for CEU samples other than NA12878 validate in long reads from NA12878. For each sample, we computed the percent of mosaic STRs classified as true positives and the significance (−log10 *P*-value) of the correlation between the mosaic fraction observed in short versus long reads, and compared those to metrics obtained for NA12878 (Supplementary Fig. S19). In all cases except the percent of true positive homopolymer calls, mosaic STRs obtained from NA12878 showed the highest validation metrics. For example, for nonhomopolymers (homopolymers), on average 29% (35%) of mosaic STRs identified in different samples were classified as potential true positives in PacBio data compared to 86% (48%) for NA12878.

### 3.4 Evaluating prancSTR on additional technologies

Finally, we evaluated the ability of prancSTR to detect mosaic STRs in two alternative technologies for which datasets were available for NA12878: Element Biosciences (Arslan *et al.* 2024) (100× coverage, 2 × 150 bp reads) and the PacBio HiFi long read dataset analyzed above. Prior to running prancSTR, we learned technology-specific error models for both technologies. Overall, we found that Element data shows far lower stutter error than the other technologies (Supplementary Table S1), including at homopolymers, whereas PacBio HiFi shows the highest error. We found that while our geometric distribution model fits Illumina error sizes well, it shows reduced fit at nonhomopolymers for Element, and further reduced for PacBio HiFi, which show higher rates of reads with errors that are not multiples of the repeat unit (Supplementary Fig. S20). Therefore, *P*-values returned by prancSTR below may not be well-calibrated for these cases and would benefit from improved technology-specific error models.

We next compared mosaic STRs identified by each technology. After filtering, a total of 4773 and 11,752 mosaic STRs were identified by Element and PacBio, respectively, compared to 411 identified by Illumina (Supplementary Table S2). When considering mosaic STRs identified by Illumina, the overall replication rate in Element is 37.7%. This rises to 93.8% (out of 16) and 39.7% (out of 312) when considering nonhomopolymer and homopolymer loci, respectively, with at least 4 reads supporting the mosaic allele (Supplementary Fig. S21), and is generally higher in cases where the germline allele is heterozygous (Supplementary Fig. S21). For example, 72.7% of heterozygous homopolymer mosaic STRs identified in Illumina replicate in Element.

Mosaic STRs identified by either Element or PacBio showed lower replication rates in other technologies than those discovered in Illumina data. For Element data, replication rates in Illumina increase with the same factors (mosaic allele frequency, mosaic support, and heterozygous versus homozygous loci) as described above. Thus the low overlap may be partially explained by increased power of detection in Element compared to Illumina due to higher coverage (mean depth 79× versus 40× in prancSTR output for each) and lower stutter error rates. On the other hand, mosaic STRs identified from PacBio data showed low overall replication rates (< 5% in all categories), suggesting a high false positive rate.

## 4 Discussion

Here, we presented prancSTR, a method for genome-wide detection of somatic mosaicism at STRs from high throughput sequencing datasets. prancSTR can accurately identify mosaic STRs without the need for a matched control sample. It has highest power to detect mosaic STRs with mosaic allele fractions of approximately 10%–20% in PCR-free datasets with 30–50× coverage, but could detect reproducible mosaic STRs sites with mosaic allele fractions as low as 7%. Application of prancSTR to population-scale short read WGS for the 1000 Genomes derived from lymphoblastoid cell lines identified hundreds of mosaic STRs per cell line with broadly consistent mosaic STR patterns across populations. Validation with orthogonal long read (PacBio HiFi) data supported 80% and 48% of high-confidence mosaic STR calls at nonhomopolymers and homopolymers, respectively, at sites with sufficient long read coverage.

prancSTR is a versatile tool that can be used to detect mosaicism in a variety of settings as long as accurate stutter error parameters are available. Our results testing prancSTR on other technologies suggests it is compatible with Element Biosciences data, which shows a large reduction in stutter error rates, especially at homopolymers, compared to Illumina. Although prancSTR can be run directly on PacBio HiFi datasets, we found a low rate of replication of mosaic STRs identified in PacBio in other technologies suggesting a high rate of false positive calls. Results for both technologies will show improvement with models that more closely capture error patterns specific to those datasets, since current models show the best fit to Illumina data. prancSTR as well as the read simulation method developed here (simTR) have been packaged into our existing toolkit, TRTools (Mousavi *et al.* 2021), enabling easy integration with other TR analysis tools.

Application of prancSTR genome-wide to WGS from 460 cell lines revealed interesting patterns of mosaic STRs. Our results broadly suggest mosaic STRs identified from short reads at nonhomopolymers and at sites with germline heterozygous genotypes are most reliable, whereas homopolymers remain particularly challenging. Overall, we found an average of 73 and 565 nonhomopolymer and homopolymer mosaic STRs per cell line, corresponding to mutation rates of approximately $10^{-4}$ and $10^{-3}$ mutations per STR per sample. Intriguingly, we identified multiple cell lines from each population with outlier mutation counts, and found strong correlation between the number of mosaic STRs at homopolymers versus nonhomopolymers. This suggests some cell lines have higher rates of STR instability than others, and that these trends are present across a broad set of loci.

prancSTR currently faces multiple limitations. First, it relies on an upstream genotyper (here, HipSTR) to provide accurate germline genotype calls as input. In cases where a mosaic allele is present at high frequency, it may be indistinguishable from a germline allele and incorrectly genotyped as heterozygous, causing mosaicism to be missed. Further, particularly at loci with high stutter error rates or low coverage, a truly heterozygous site may be incorrectly genotyped as homozygous, causing prancSTR to incorrectly identify the second germline allele as mosaicism. As a result, mosaic STRs identified at heterozygous sites are likely more reliable. Second, prancSTR currently focuses on identifying mosaic STRs with a single high frequency mosaic allele. While this is likely to capture mosaic events at shorter STRs, longer repeats such as the Huntington’s Disease locus where mosaicism is known to play a role in disease pathogenesis tend to show a broad range of mosaic allele lengths (Swami *et al.* 2009). Still, simulations show that the method is already able to detect more complex cases with multiple mosaic alleles, with similar power compared to single mosaic allele scenarios. Third, similar to mosaicism detection tools for other variant types, prancSTR is limited by the coverage of current datasets, which is insufficient to detect most mosaic events below 5% frequency.

Overall, prancSTR can serve as a valuable method to characterize somatic mosaicism at STRs in healthy individuals or in disease settings such as microsatellite instability in cancer (Boland and Goel 2010) or neurological diseases (Telenius *et al.* 1994) where mosaicism is known to play a key role. We envision multiple future extensions of this framework. prancSTR’s model can be extended to directly model cases with multiple mosaic alleles. Further, incorporating phase information from haplotagged reads can help determine if a mosaic allele is found on a single haplotype, and can therefore be used to distinguish heterozygous versus high frequency mosaic alleles. Long reads, which can be easily haplotagged, are an especially promising solution to this challenge. Finally, a combination of improved STR error models as well as the steady reduction of error rates is likely to improve detection of mosaic STRs directly from long reads in the future.

## Supplementary Material

btae485_Supplementary_Data

## Data Availability

The data underlying this article are available in the article and in its online supplementary material.
