# Supplementary material for: Genome-wide detection of somatic mosaicism at short tandem repeats
Source: Bioinformatics. 2024 Jul 30;40(8):btae485. doi: 10.1093/bioinformatics/btae485 (PMC11319640; doi:10.1093/bioinformatics/btae485)
Supplement: btae485_Supplementary_Data [file btae485_supplementary_data.zip › BIOINF-2023-2274-SupplementaryFigures.pdf]

Supplementary Material - prancSTR

Supplementary Figures

Supplementary Figure 1

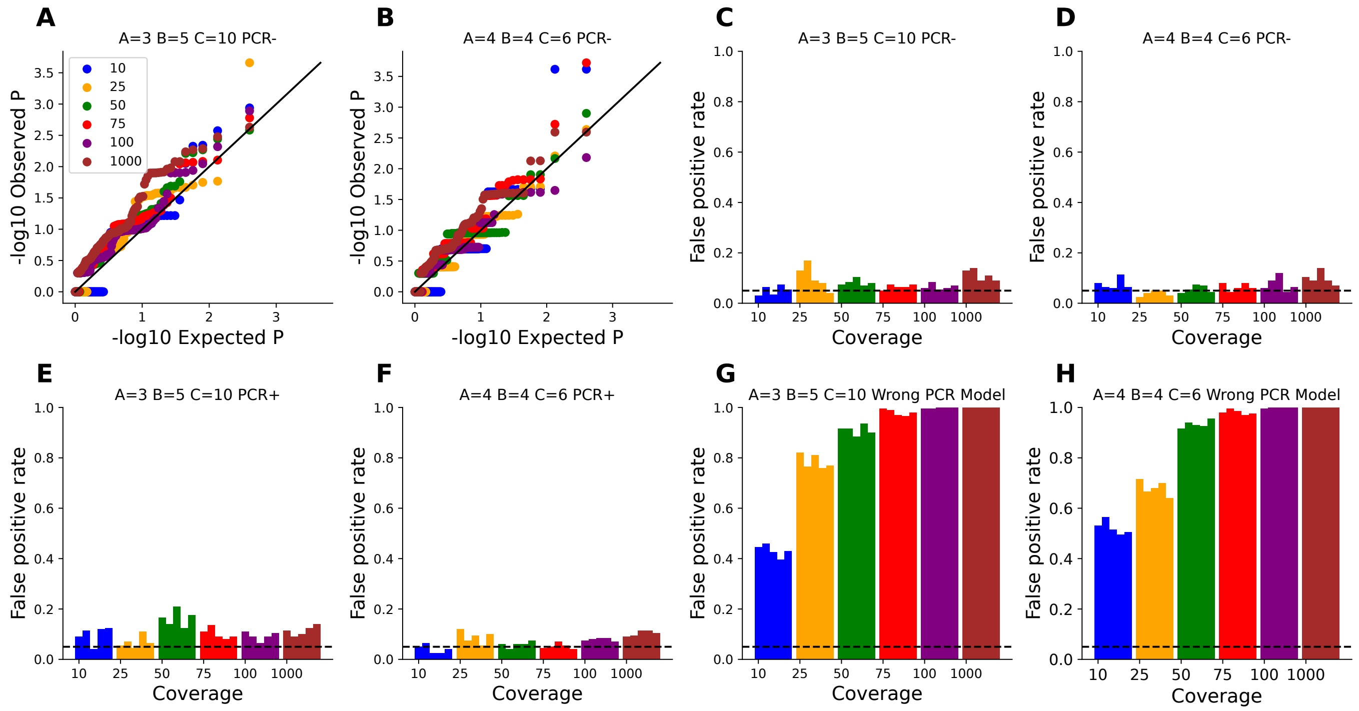

prancSTR P-values are well-calibrated. (A-B) represent quantile-quantile (QQ) plots showing the distribution of P-values in simulations where the germline genotype is either heterozygous (A) or homozygous (B) under different coverage levels (denoted by dot colors) and where no mosaicism was simulated ($f = 0$). As expected, P-values follow the uniform distribution. (C-D) show the false positive rate, computed as the percent of null ($f = 0$) simulations (n=200) in which a significant P-value ($p<0.05$) was obtained. A-D use a PCR-free error model with u=0.02, $d = 0.02$, ρ=0.9. (E-F) are similar to (C-D) but are based on a PCR+ error model (u=0.1, d=0.1, ρ=0.9). (G-H) are similar to (C-D) but we used a PCR+ error model to simulate data but used a PCR-free model for mosaicism detection with prancSTR. G-H illustrate the importance of using accurate stutter models for mosaicism identification. Panels here are based on simulated read vectors. For C-H, bar color denotes coverage. For each coverage level, to assess potential variability across runs, we performed 5 different sets of 200 simulations, each time varying the seed used for random number generation.

Supplementary Figure 2

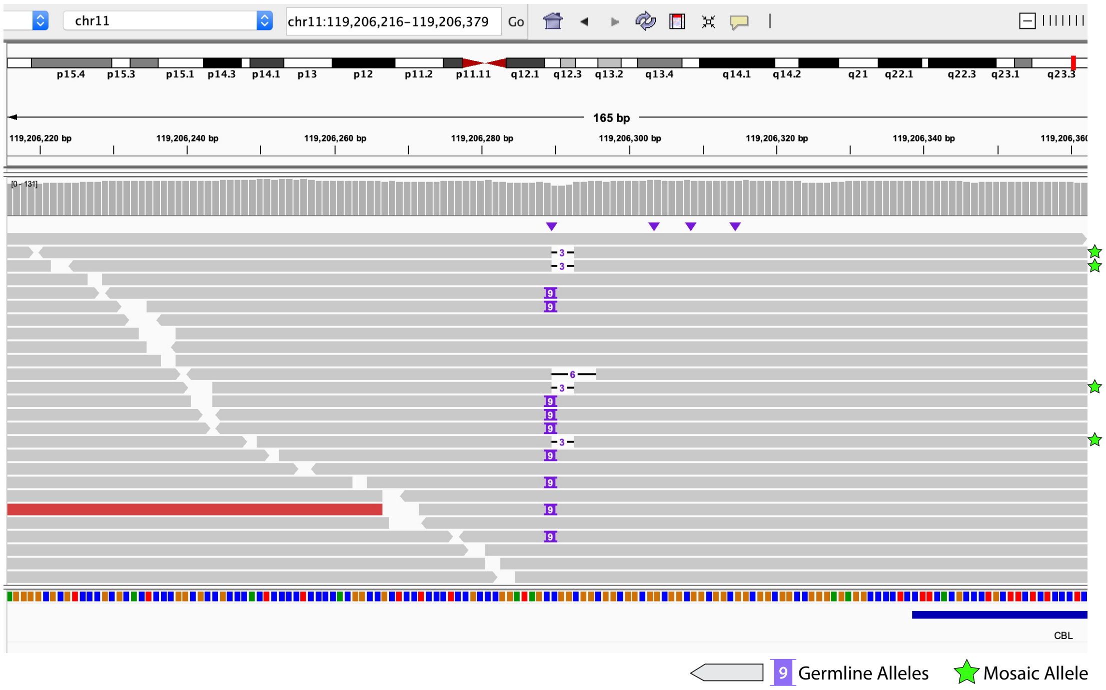

Visualization of reads simulated by simTR. Reads were simulated in a 1000bp window around a CGG repeat in *CBL* (hg38 chr11:119206289-119206322). The simulation was based on a germline genotype of 11 (reference allele; denoted by gray bars in the repeat region) and 14 copies (reference + 9bp; denoted by purple rectangles with “9”) of the repeat, and a mosaic allele with 10 copies (reference - 3bp) at 10% variant allele fraction. The visualization was produced using the Integrative Genomics Viewer (https://software.broadinstitute.org/software/igv/).

Supplementary Figure 3

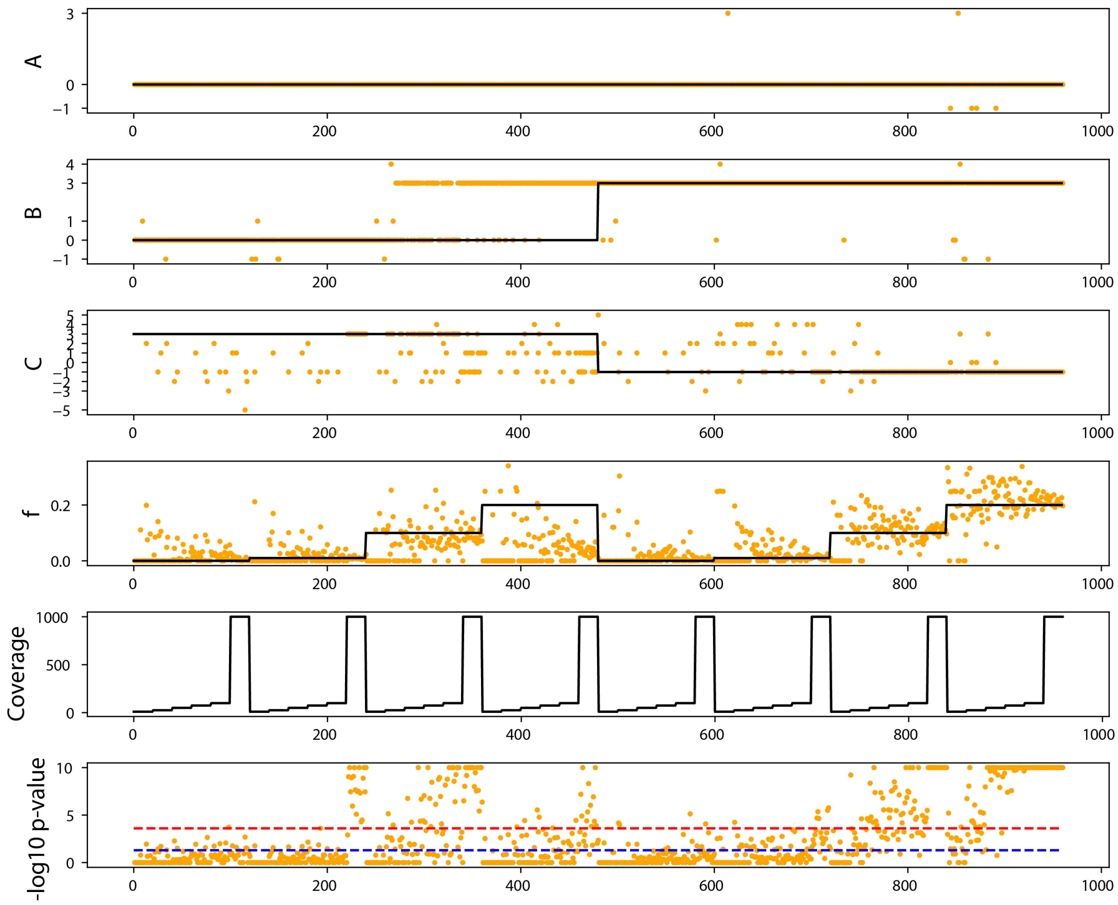

Benchmarking prancSTR using simulated reads - CBL locus. We used simTR to simulate reads which were input to HipSTR followed by prancSTR to detect STR mosaicism under a range of settings. Results are shown for a CGG repeat near *CBL* (hg38 chr11:119206289-119206322). We varied the germline genotypes (A and B; top two panels), mosaic allele (C; third panel), mosaic allele fraction (f; fourth panel), and target coverage (fifth panel). In each of the top five panels, black lines show the simulated value. Each simulation setting was performed 20 times. Orange dots denote estimated parameter values. Germline genotypes (A and B) are those estimated by HipSTR. Estimated values of f and C are obtained from prancSTR. The bottom plot shows the -log10 P-value obtained in each case by prancSTR, which tests the null hypothesis that $f = 0$. The blue dashed line denotes P=0.05. The red dashed line denotes P=0.00025, which is approximately the genome-wide significance threshold used to identify mosaic STRs at FDR 5% when running on a single sample (NA12878).

Supplementary Figure 4

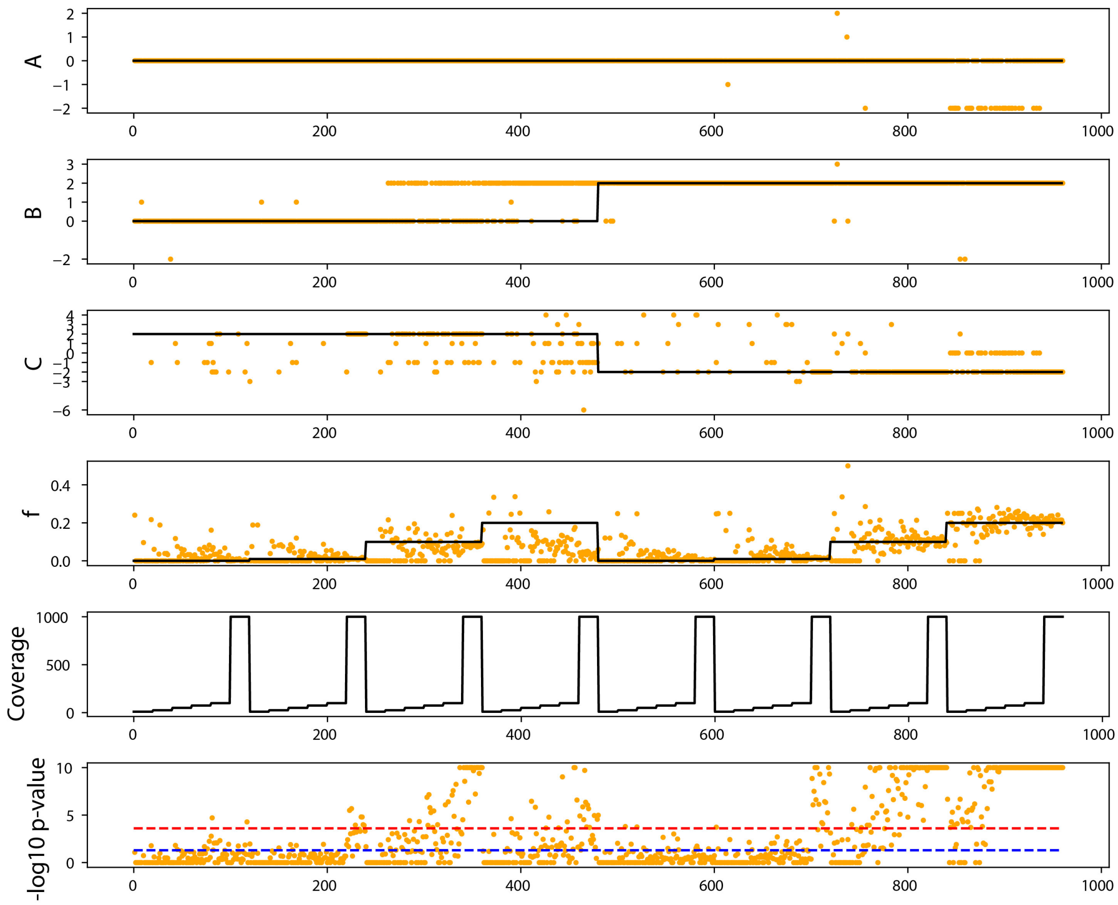

Benchmarking using simulated reads - CSF1PO locus. Panels are same as in **Supplementary Fig. 3** except reads are simulated for the tetranucleotide (AGAT) CSF1PO locus (hg38 chr5:150076324-150076375).

Supplementary Figure 5

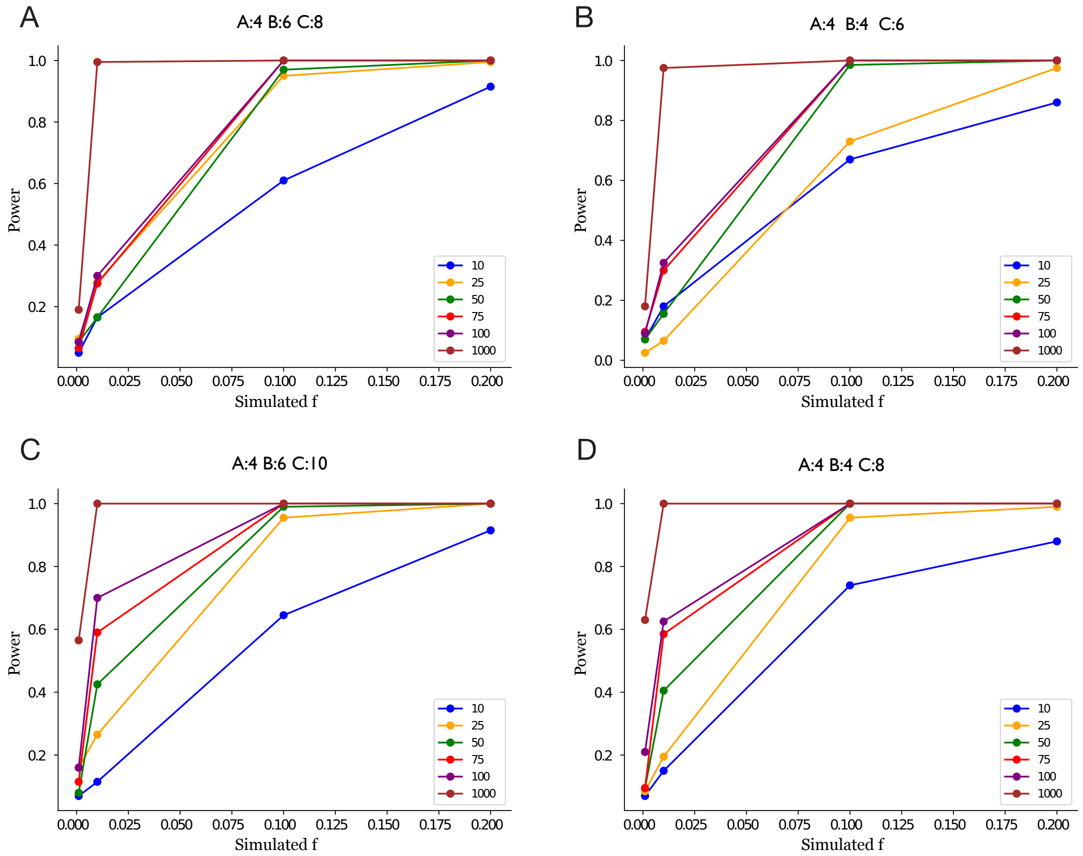

Power of detection across different values of C. (A-B) show the power of detection in a germline heterozygous (A) vs homozygous (B) case, where the mosaic allele C is two repeat units away from the nearest germline allele. (C-D) show the power of detection in a heterozygous (C) vs homozygous (D) case, where the mosaic allele C is four repeat units away from nearest germline allele. As in **Fig. 1D-E**, power is computed as the percent of simulations for which $P < 0.05$. Lines denote different coverage levels, where coverage gives the total number of reads spanning the STR of interest. Panels here are based on simulated read vectors.

Supplementary Figure 6

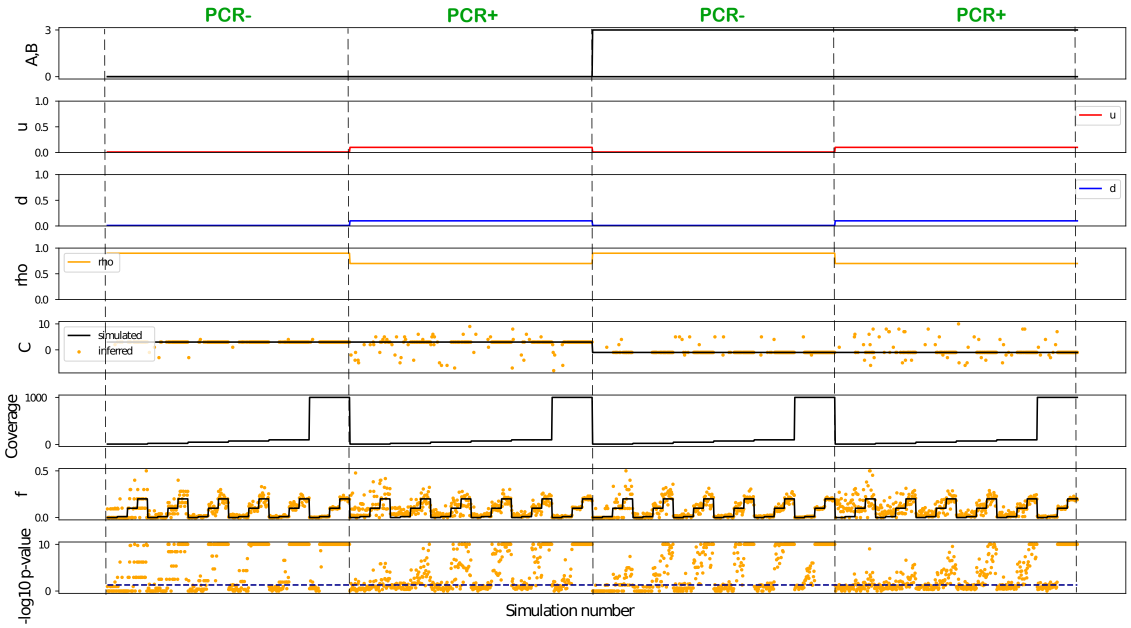

Evaluating mosaic STR detection under additional simulation settings. The top seven panels show simulated values for the germline genotype (A, B; top panel), stutter expansion probability (u; second panel), stutter contraction probability (d; third panel), stutter step size (rho; fourth panel), mosaic allele (C; fifth panel), coverage (sixth panel), and mosaic allele fraction (f; seventh panel). In each of the top seven panels, the lines shows the simulated value. Each simulation setting was performed 20 times. Orange dots denote estimated parameter values. The bottom plot shows the -log10 P-value obtained in each case by prancSTR, which tests the null hypothesis that $f = 0$. The dark blue dashed line denotes P=0.05. Panels here are based on simulated read vectors.

Supplementary Figure 7

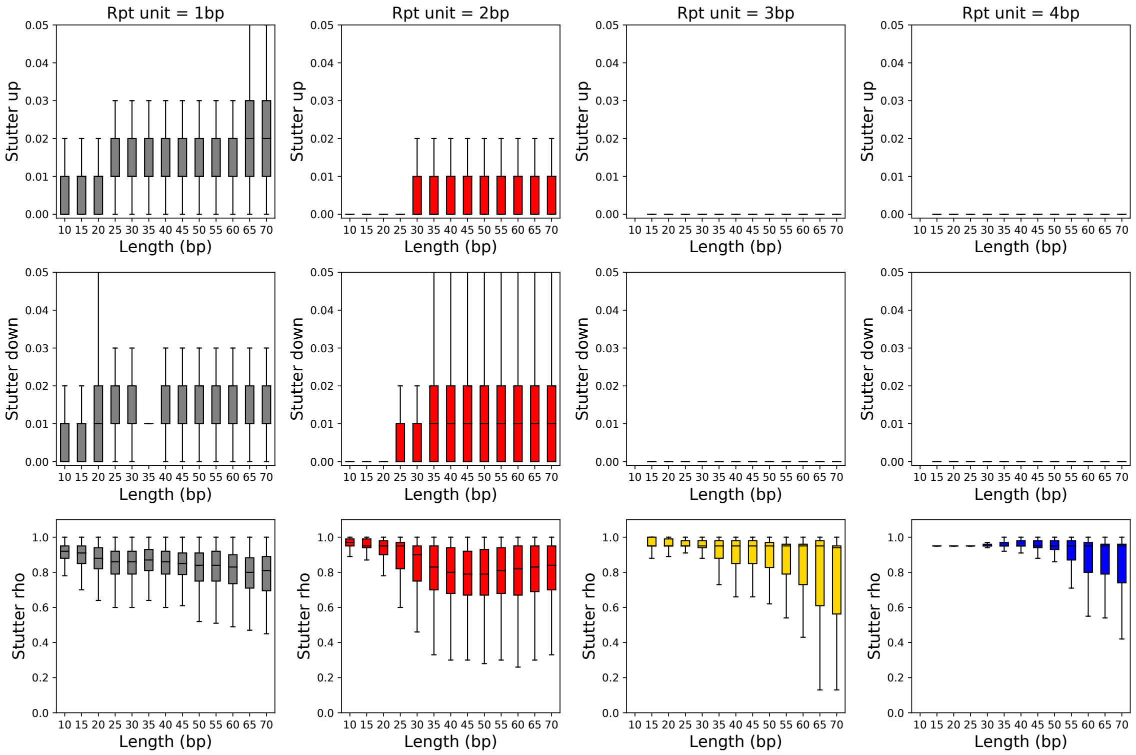

Characterizing stutter parameters in the 1000 Genomes. Per-locus stutter parameters were previously estimated from PCR-free high-coverage WGS for 1000 Genomes samples using HipSTR (Ziaei Jam *et al.*, 2023). Boxplots in each row show the distribution of the three stutter parameters output by HipSTR (top=INFRAME_UP, middle=INFRAME_DOWN; bottom=INFRAME_PGEOM) for STRs with different repeat unit lengths (columns: gray=homopolymers; red=dinucleotides; gold=trinucleotides; blue=tetranucleotides). Horizontal lines show median values, boxes span from the 25th percentile (Q1) to the 75th percentile (Q3). Whiskers extend to Q1–1.5*IQR (bottom) and Q3+1.5*IQR (top), where IQR gives the interquartile range (Q3-Q1).

Supplementary Figure 8

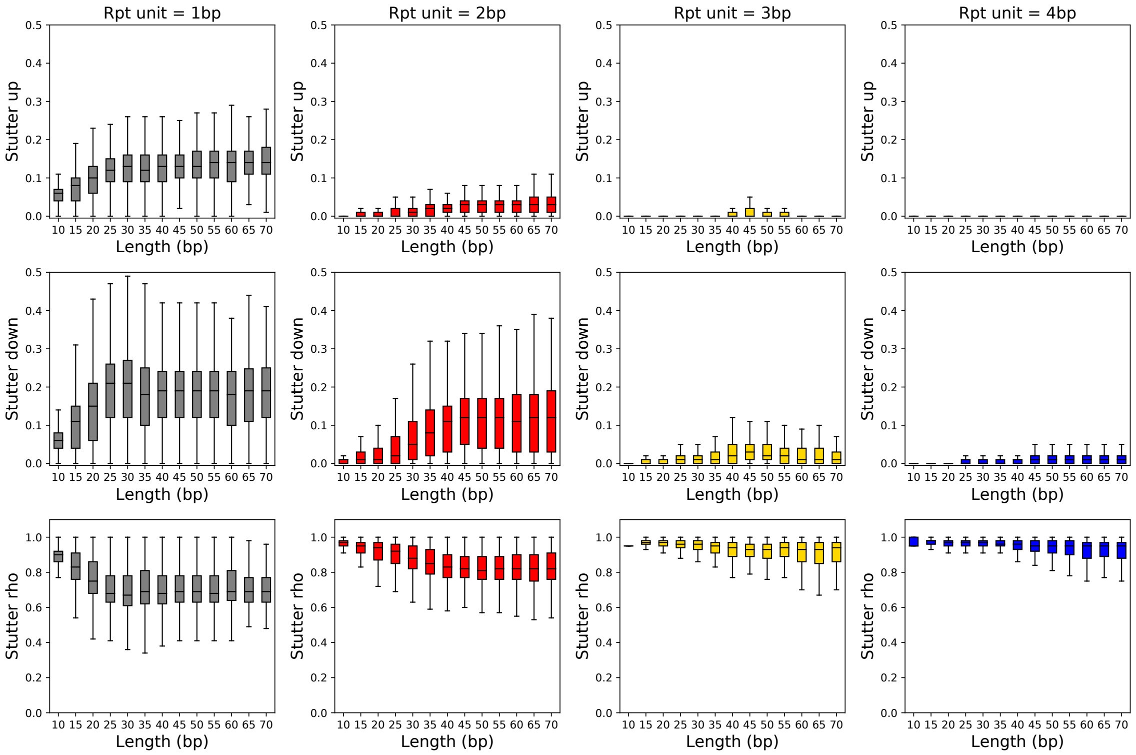

Characterizing stutter parameters in H3Africa. Per-locus stutter parameters were previously estimated from PCR+ high-coverage WGS for individuals from the H3Africa cohort using HipSTR (Ziaei Jam *et al.*, 2023). Panels showing distributions of stutter parameters are the same as in **Supplementary Fig. 7** except based on H3Africa data.

Supplementary Figure 9

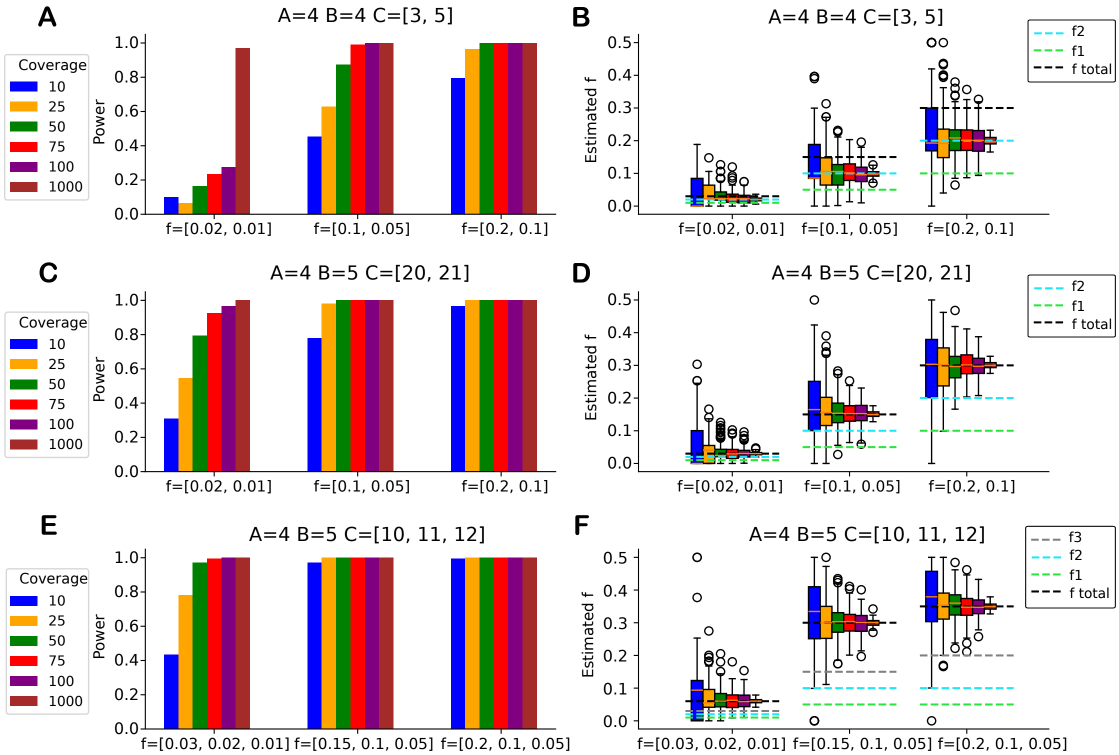

Mosaic STR detection using prancSTR in simulated data with multiple mosaic alleles. Each row shows a different simulation scenario, with the values for the diploid genotype (A and B) and list of mosaic alleles (C) annotated at the top of each plot. For each setting, we simulated mosaic STRs with different allele frequencies for the mosaic alleles. Left: barplots show the power to detect mosaic STRs under each setting. Colors denote coverage. Power is based on the percentage out of 200 simulations for which prancSTR returned a P-value < 0.05. Right: boxplots show the distribution of the value of f inferred by prancSTR across all 200 simulations for each setting. Color denotes coverage. Colored dashed horizontal lines show the simulated frequencies for each of the different mosaic alleles. Black dashed horizontal lines show the total simulated frequency of all mosaic alleles.

Supplementary Figure 10

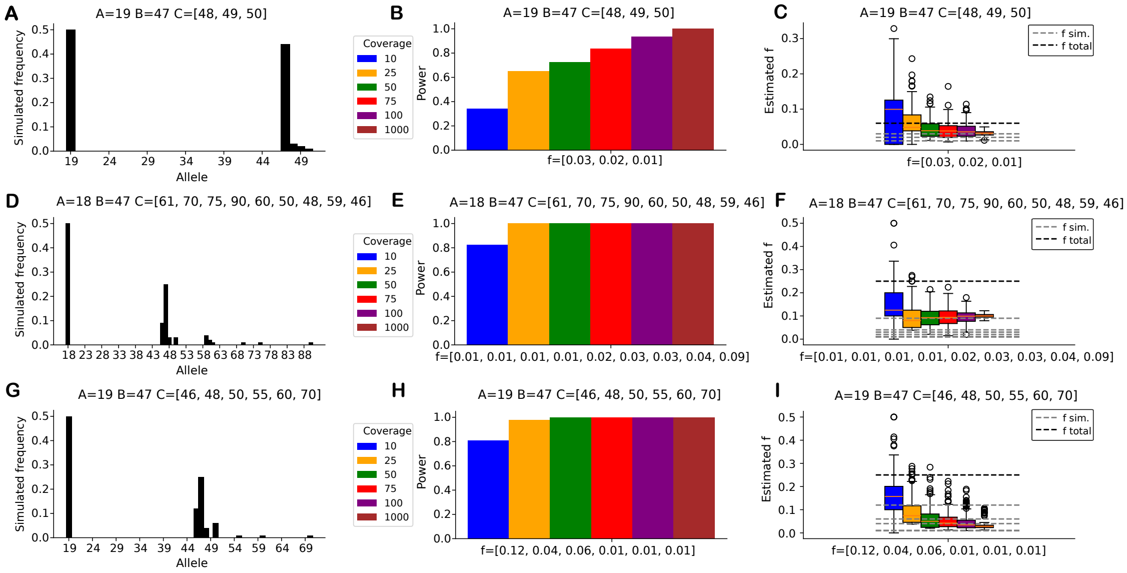

Mosaic STR detection using prancSTR in simulated data representing realistic Huntington's scenarios. Each row shows a different simulation scenario, with the values for the diploid genotype (A and B) and list of mosaic alleles (C) annotated at the top of each plot. Left: histograms show the simulated allele frequencies of each allele (both germline and mosaic alleles). Middle: barplots show the power to detect mosaic STRs under each setting. Colors denote coverage. Power is based on the percentage out of 200 simulations for which prancSTR returned a P-value < 0.05. Right: boxplots show the distribution of the value of f inferred by prancSTR across all 200 simulations for each setting. Color denotes coverage. Gray dashed horizontal lines show the simulated frequencies for each of the different mosaic alleles. Black dashed horizontal lines show the total simulated frequency of all mosaic alleles.

Supplementary Figure 11

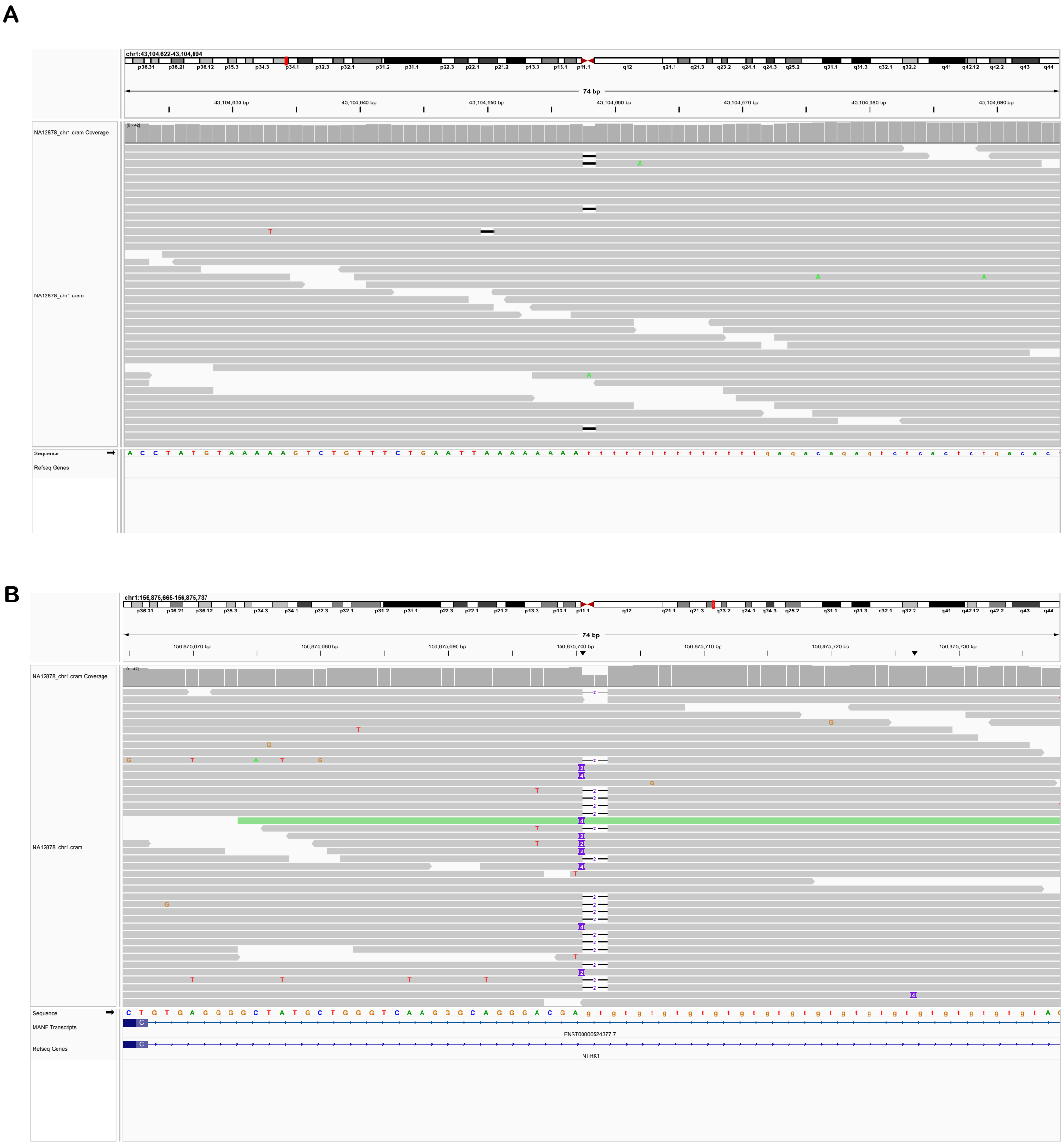

Example mosaic STRs detected by prancSTR in NA12878 as observed in IGV. The snapshots provide examples of read evidence for mosaic STRs detected in NA12878 by prancSTR. Reads were extracted from the NA12878 cram file corresponding to a 300bp window centered at the target STRs. (A) Gives an example of a homopolymer T repeat where prancSTR detected mosaicism. The germline genotype was called as homozygous for the reference allele. prancSTR inferred a mosaic allele with 1 deletion (1 less repeat copy and 1 bp deletion as compared to the reference). (B) For this example, showing a dinucleotide GT repeat, the germline genotype was called as heterozygous for -1 and +2 copies of GT compared to the reference genome (corresponding to a deletion of 2 bp and an insertion of 4bp, respectively, relative to the reference). prancSTR inferred a mosaic allele of 1 additional copy (a 2bp insertion compared to the reference). Visualizations were produced using the Integrative Genomics Viewer (https://software.broadinstitute.org/software/igv/).

Supplementary Figure 12

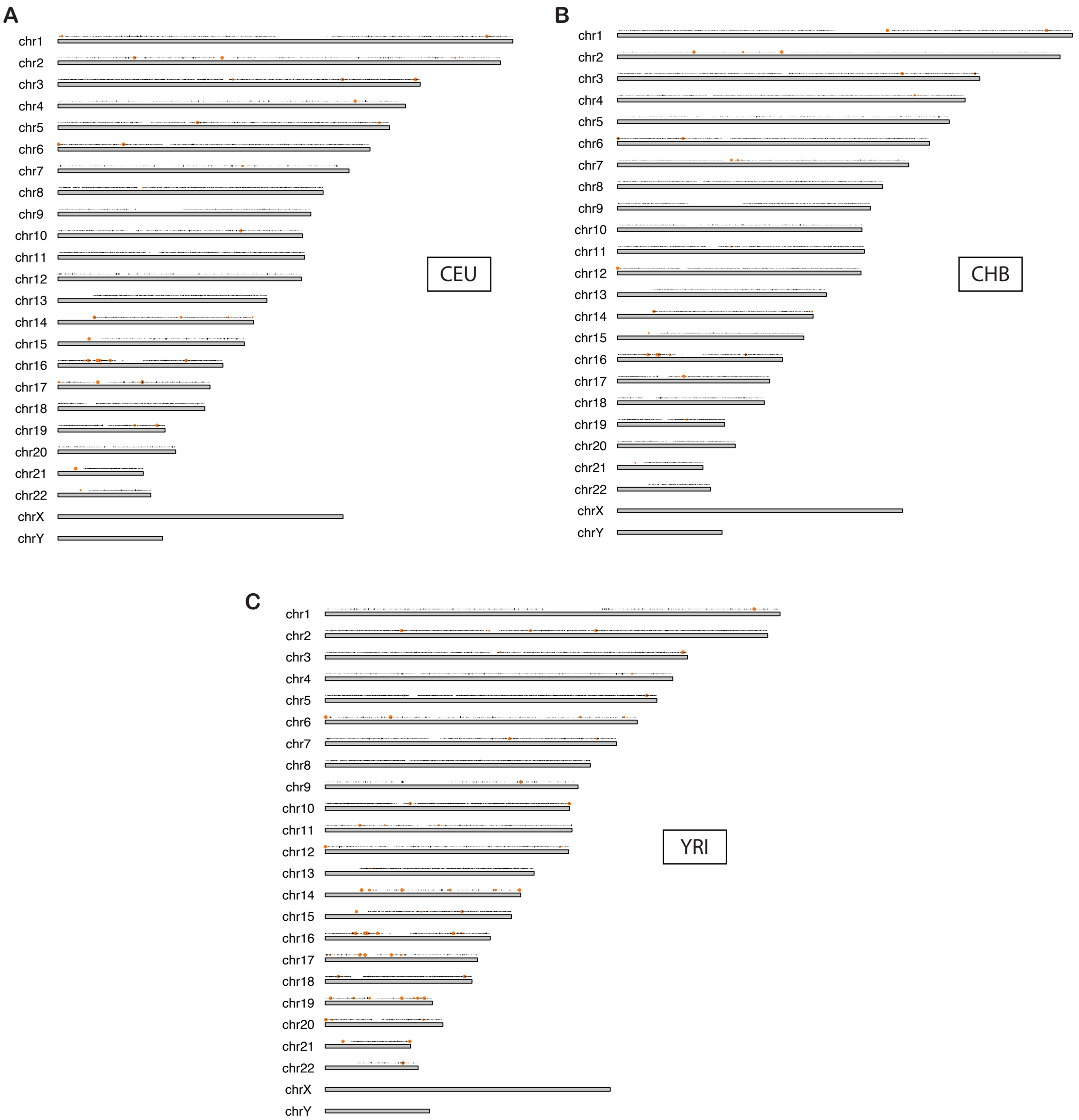

Karyograms showing locations of mosaic STRs. Karyograms are shown for each population (A=CEU, B=CHB, C=YRI). Each black dot indicates the location of a unique mosaic STR. Dot size scales with the number of unique samples with a mosaic STR at that site. Orange dots indicate mosaic STRs called in more than 10 samples in each population.

Supplementary Figure 13

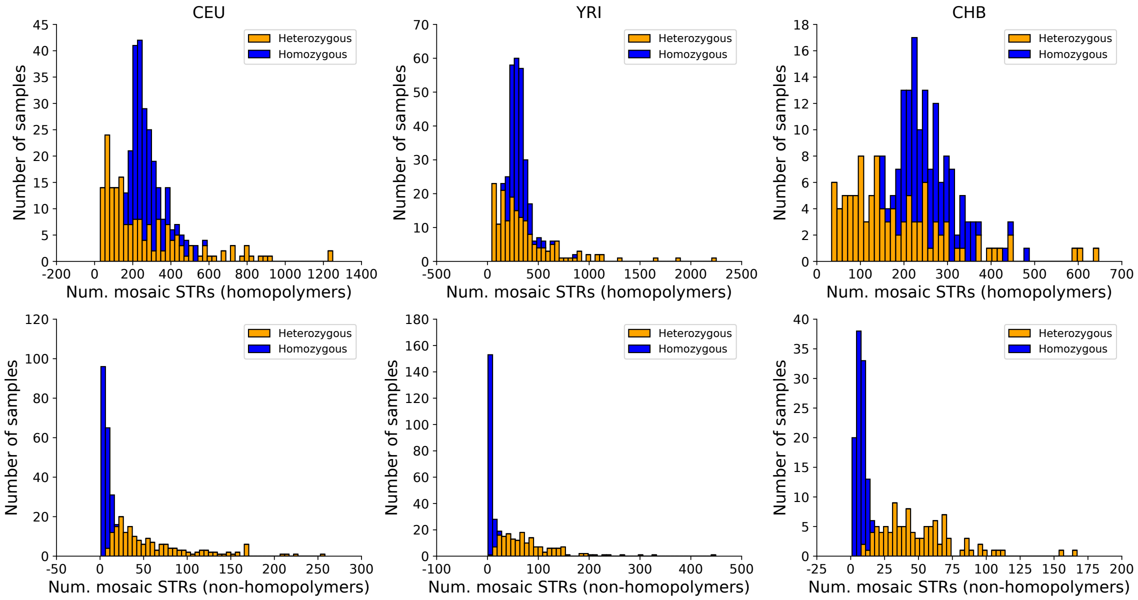

Distribution of the number of mosaic STRs detected per sample in each population Stacked histograms show the number of mosaic STRs detected per cell line after quality filtering (**Methods**). Blue=mosaic STRs at germline homozygous sites and orange=mosaic STRs at germline heterozygous sites. Top and bottom plots show counts at homopolymer and non-homopolymer STRs, respectively. Data is shown separately for CEU (left), YRI (middle), and CHB (right).

Supplementary Figure 14

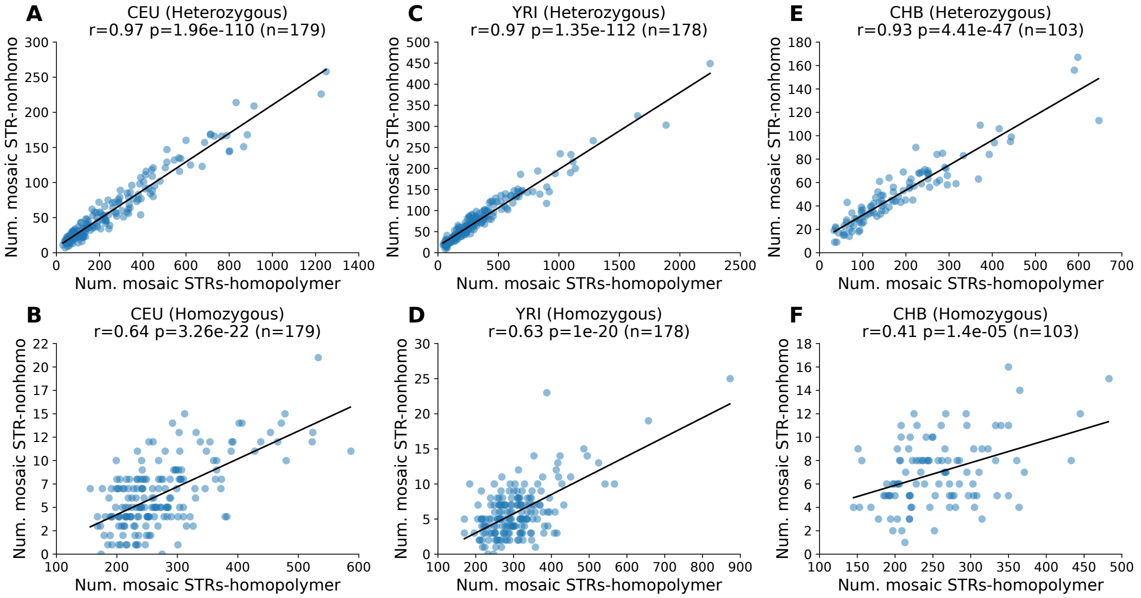

Distribution of mosaic STRs detected per sample for homopolymers vs non-homopolymers. Scatter plots show the number of mosaic STRs detected per cell line after quality filtering (**Methods**) in homopolymers (x-axis) vs non-homopolymers (y-axis). Top and bottom plots are restricted to mosaic STRs occurring at germline heterozygous and homozygous sites, respectively. Data is shown separately for CEU (left), YRI (middle), and CHB (right). Black lines show the best linear fit. Pearson correlation coefficients and corresponding two-sided p-values are annotated in each plot.

Supplementary Figure 15

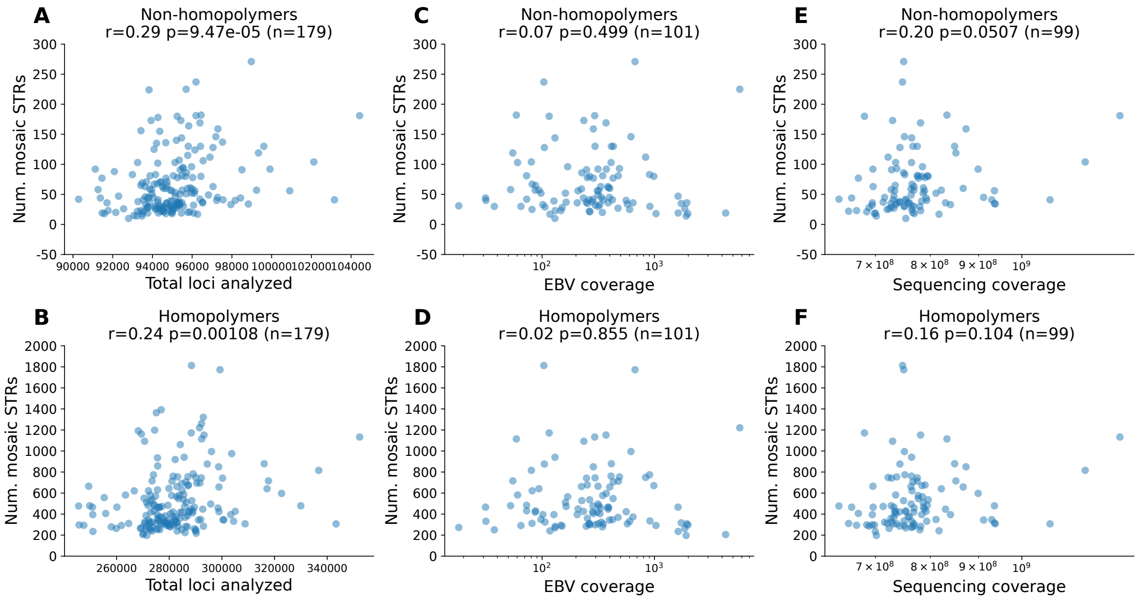

Evaluation of factors influencing the number of mosaic STRs per cell line. Scatter plots show the relationship between various factors (x-axis) and the number of mosaic STRs detected after filtering (y-axis). (A-B) Total number of loci analyzed per cell line, (C-D) EBV coverage, (E-F) sequencing coverage. Top and bottom plots are restricted to mosaic STRs occurring at non-homopolymers and homopolymers, respectively. Data is shown for CEU only. Other populations showed similar trends (not shown). Pearson correlation coefficients and corresponding two-sided P-values are annotated in each plot.

Supplementary Figure 16

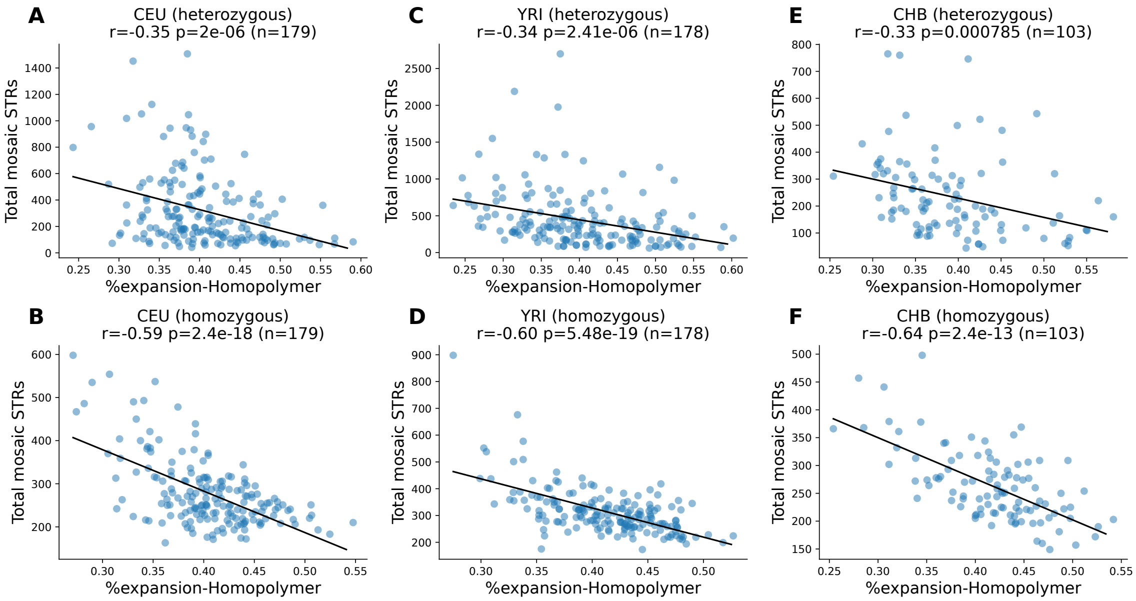

Relationship between expansion bias and number of mosaic STRs per cell line. Scatter plots show the % of mosaic STRs that are expansions (x-axis) vs. the total number of mosaic STRs detected per cell line (y-axis). Top and bottom plots are restricted to mosaic STRs occurring at germline heterozygous and homozygous sites, respectively. Data is shown separately for CEU (left), YRI (middle), and CHB (right). Black lines show the best linear fit. Pearson correlation coefficients and corresponding two-sided P-values are annotated in each plot. Data is only shown for homopolymers.

Supplementary Figure 17

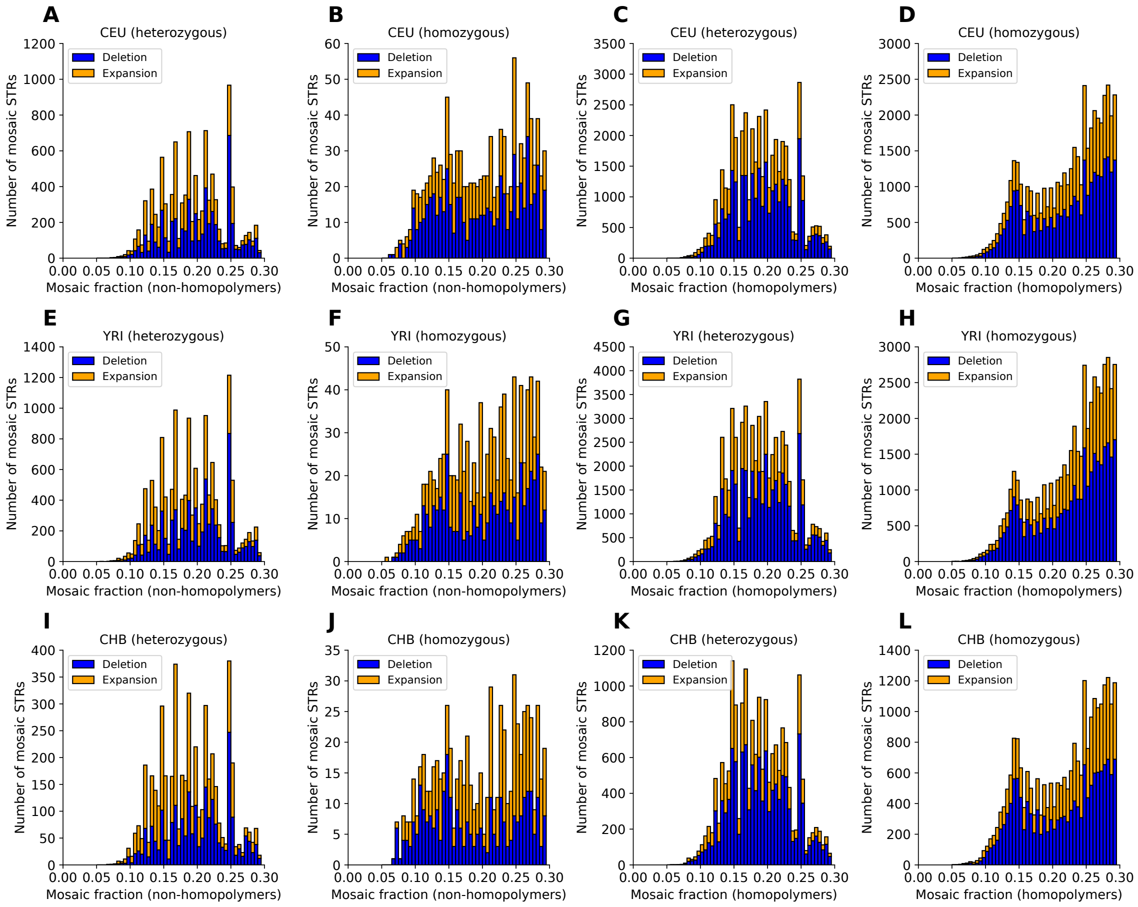

Distribution of f across all mosaic STRs detected in 1000 Genomes cell lines. Left two and right two plots in each row are restricted to mosaic STRs occurring at non-homopolymers and homopolymers respectively. The first and third plot in each column are restricted to heterozygous sites and second and fourth show homozygous sites. The histograms are further broken down by expansions (orange) and deletions (blue). Bars are stacked, such that the y-axis value of each bar denotes the total number of mosaic STRs falling in each bin of f-values. Rows show data for CEU (A-D), YRI (E-H), and CHB (I-L).

Supplementary Figure 18

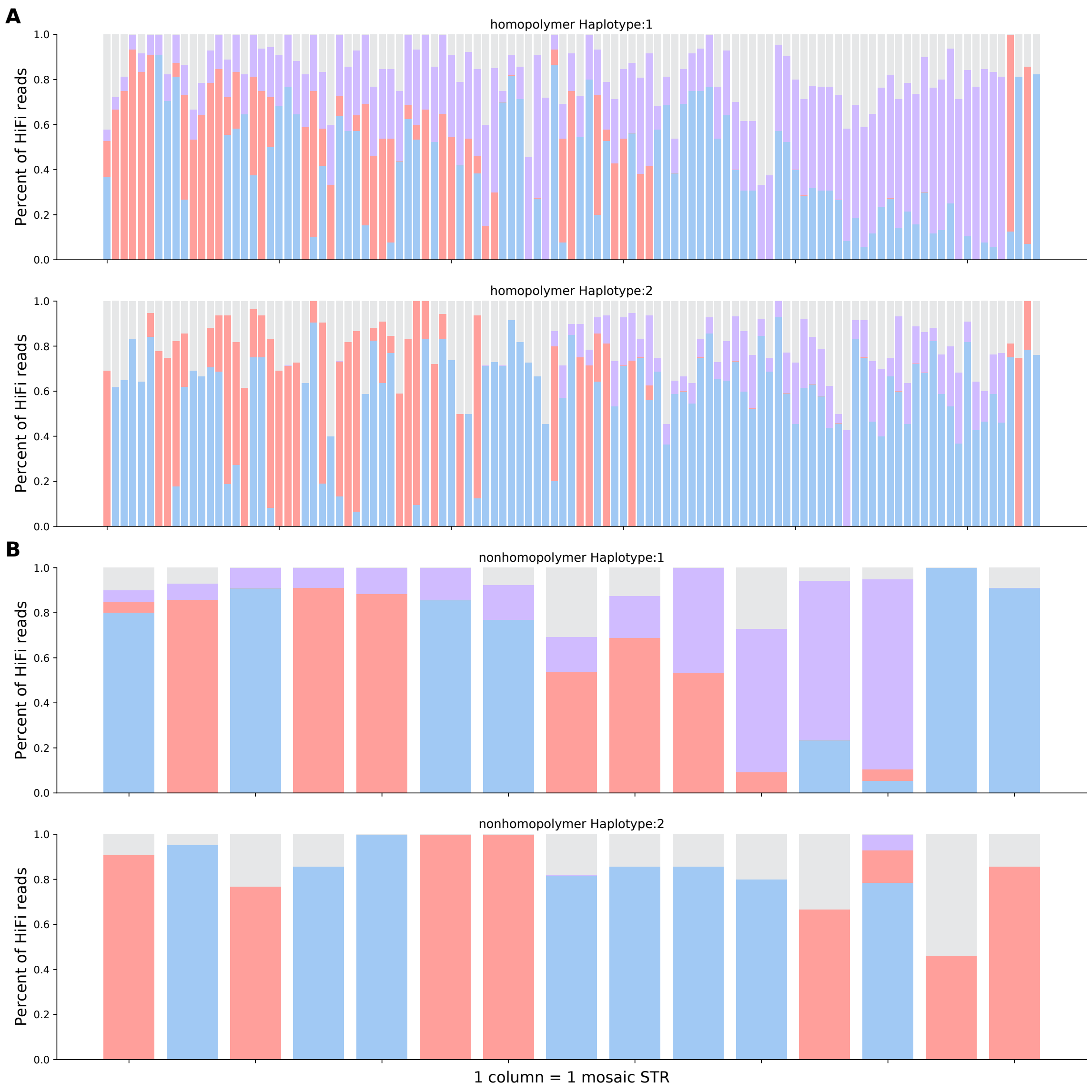

Inspection of per-locus long read support for candidate mosaic STRs inferred from short reads in NA12878. Each bar shows the percentage of PacBio HiFi reads on each haplotype supporting the germline genotype alleles (scarlet and blue), mosaic allele (lavender), or other alleles (gray). (A) shows the two haplotypes at each candidate non-homopolymer mosaic STR and (B) shows the two haplotypes at homopolymer mosaic STRs. The order of the two haplotypes at each locus is arbitrary.

Supplementary Figure 19

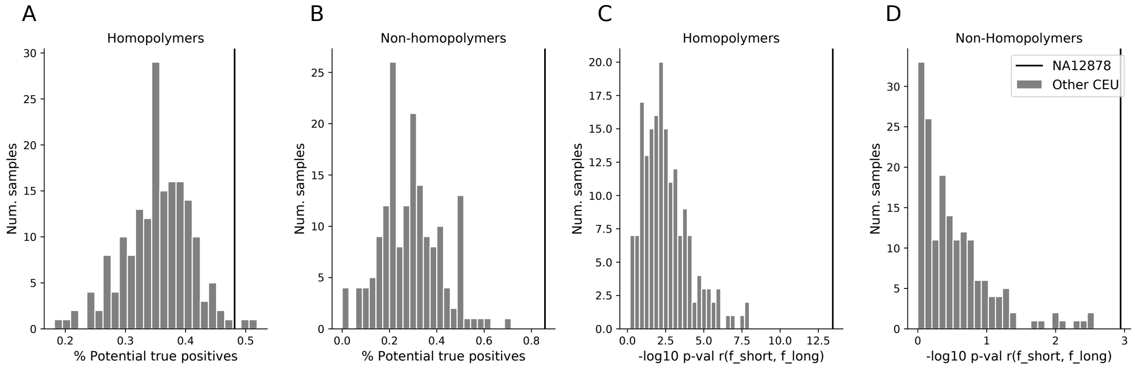

Comparison of NA12878 long read validation metrics in other CEU samples. As a negative control, we performed long read validation analysis using mosaic STRs identified in other CEU samples from short reads and compared metrics to those obtained from using short reads for the sample matching the long read data (NA12878). Histograms (gray bars) show the distribution of each metric in all CEU samples except NA12878. The black line indicates the metric computed in NA12878. (A) and (B) show the percentage of short read mosaic STRs identified as potential true positives in long reads for homopolymer and non-homopolymer loci, respectively. (C) and (D) show the -log10 p-value of the Pearson correlation between the mosaic allele fraction estimated from short reads vs. that observed in long reads for homopolymer and non-homopolymer loci, respectively.

Supplementary Figure 20

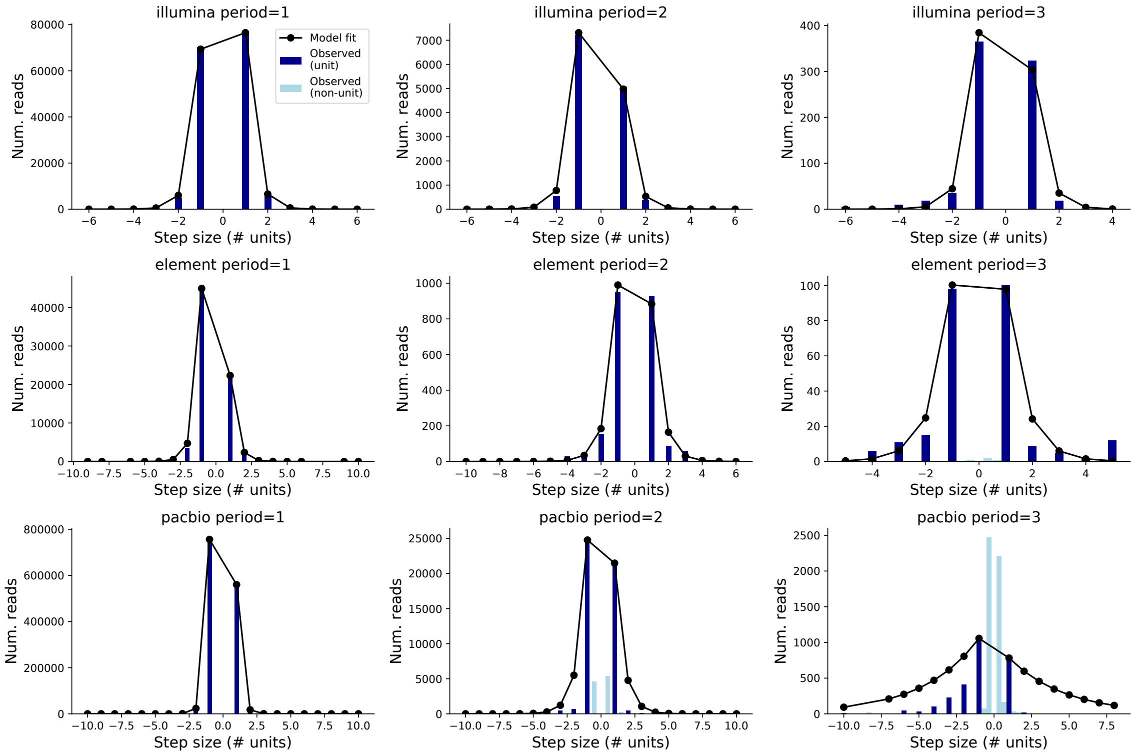

Stutter model fit across different technologies. Each panel shows the distribution of error step sizes for different repeat unit lengths (left=homopolymers, middle=dinucleotides, right=trinucleotides) and technologies (top=Illumina, middle=Element, bottom=Pacbio). The x-axis gives the error size in terms of repeat units away from the true genotype. Blue bars show the number of reads with each observed error size. Dark blue indicates step sizes are in complete copies of the repeat unit, and light blue indicates non-unit step sizes. Black lines show the expected read counts at each error size based on inferred stutter models.

Supplementary Figure 21

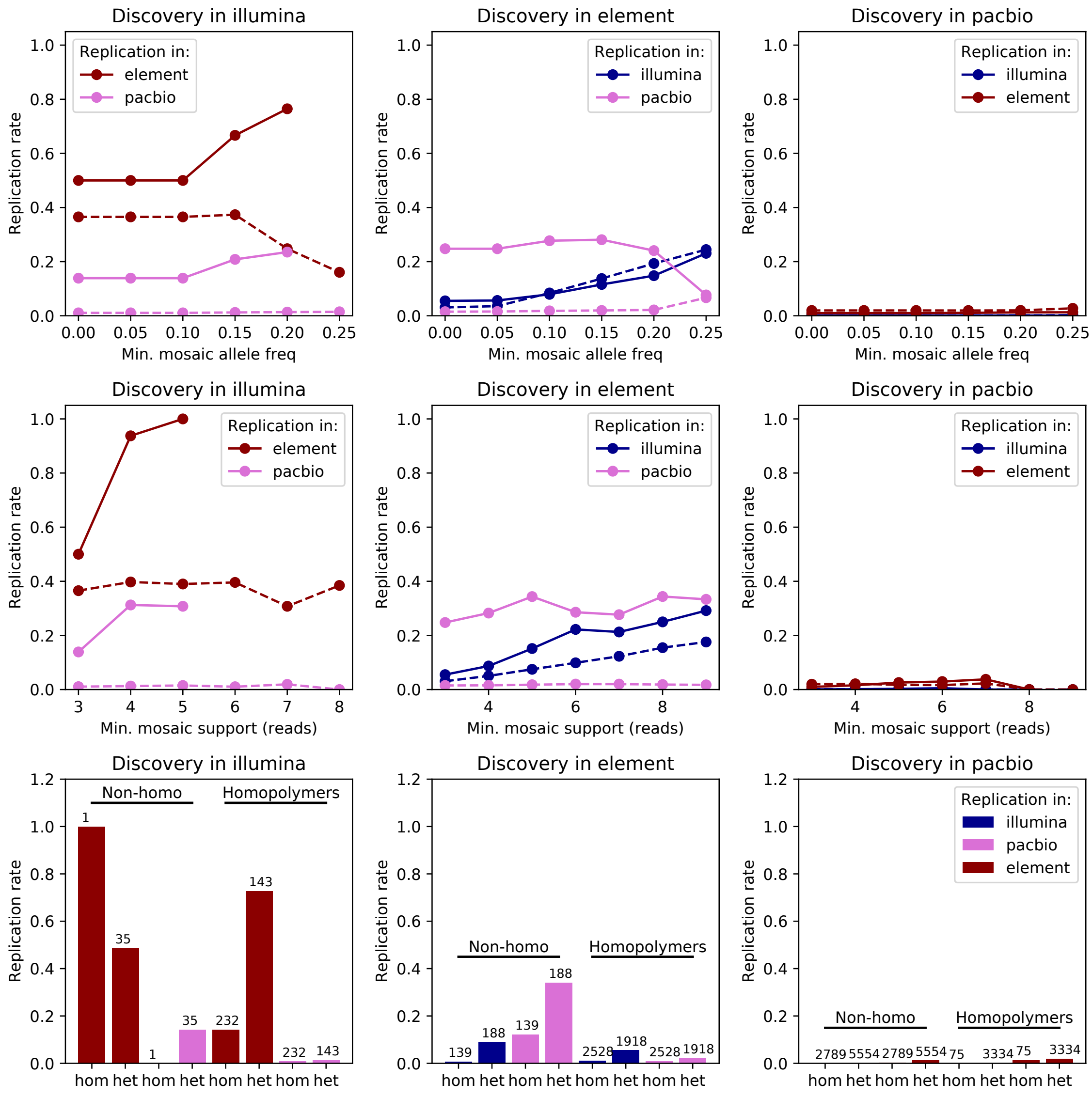

Comparison of mosaic STRs identified across technologies. Each column describes replication rates of mosaic STRs discovered by each technology (left=Illumina, middle=Element, right=Pacbio) Top row: the x-axis shows the minimum mosaic allele frequency (f) and the y-axis shows the replication rate, defined as the percentage of discovery mosaic STRs also identified by the other two technologies. Maroon=replication in Element; light purple=replication in PacBio; blue=replication in Illumina. Solid lines are for non-homopolymer and dashed lines are for homopolymer STRs, respectively. Data points are only shown for categories with at least 10 mosaic STRs. Middle row: the x-axis shows the minimum number of reads supporting the mosaic allele in the discovery dataset. The y-axis shows replication rate. Colors are same as in the top row. Bottom row: replication rates are shown when considering STRs with homozygous vs. heterozygous germline genotypes, and are further stratified by homopolymer vs. non-homopolymer loci. Annotations above each bar show the number of mosaic STRs considered in each category.

Supplementary Tables

Supplementary Table 1

Category	Technology	Numrecords	Numreads	u	d	ρ
Homopolymer	Illumina PCR-free	485420	12172773	0.006877	0.006235	0.914
	PacBio HiFi	461984	7873224	0.073434	0.099146	0.969
	Element	497632	27580749	0.000909	0.001830	0.895
Dinucleotide	Illumina PCR-free	185704	4581606	0.001214	0.001785	0.894
	PacBio HiFi	179268	3083610	0.008973	0.010351	0.778
	Element	187127	9569386	0.000117	0.000131	0.814
Trinucleotide	Illumina PCR-free	61938	1532528	0.000224	0.000284	0.884
	PacBio HiFi	59127	1014833	0.003265	0.004414	0.237
	Element	61704	3170712	0.000041	0.000042	0.753
Other	Illumina PCR-free	308693	7335291	0.000048	0.000155	0.781
	PacBio HiFi	291732	4982824	0.004704	0.013949	0.123
	Element	310041	15753521	0.000020	0.000045	0.661

Inferred stutter model parameters for each technology. Parameters were inferred using high confidence homozygous calls from NA12878 as ground truth genotypes. Numrecords and Numreads indicate the number of unique STRs and total number of reads used to estimate each model, respectively. u and d give the probability for a read to show an insertion or deletion error, respectively. ρ is the parameter of a geometric distribution fit to the error sizes. The stutter model parameters are described in detail in the Methods.

References

Ziaei Jam, H. et al (2023). A deep population reference panel of tandem repeat variation. *Nat Commun*, **14**(1), 6711.
